# A comprehensive investigation of clustering algorithms for User and Entity Behavior Analytics

**DOI:** 10.3389/fdata.2024.1375818

**Published:** 2024-05-09

**Authors:** Pierpaolo Artioli, Antonio Maci, Alessio Magrì

**Affiliations:** Cybersecurity Laboratory, BV TECH S.p.A., Milan, Italy

**Keywords:** clustering, data analytics, machine learning, UEBA, unsupervised learning

## Abstract

**Introduction:**

Government agencies are now encouraging industries to enhance their security systems to detect and respond proactively to cybersecurity incidents. Consequently, equipping with a security operation center that combines the analytical capabilities of human experts with systems based on Machine Learning (ML) plays a critical role. In this setting, Security Information and Event Management (SIEM) platforms can effectively handle network-related events to trigger cybersecurity alerts. Furthermore, a SIEM may include a User and Entity Behavior Analytics (UEBA) engine that examines the behavior of both users and devices, or entities, within a corporate network.

**Methods:**

In recent literature, several contributions have employed ML algorithms for UEBA, especially those based on the unsupervised learning paradigm, because anomalous behaviors are usually not known in advance. However, to shorten the gap between research advances and practice, it is necessary to comprehensively analyze the effectiveness of these methodologies. This paper proposes a thorough investigation of traditional and emerging clustering algorithms for UEBA, considering multiple application contexts, i.e., different user-entity interaction scenarios.

**Results and discussion:**

Our study involves three datasets sourced from the existing literature and fifteen clustering algorithms. Among the compared techniques, HDBSCAN and DenMune showed promising performance on the state-of-the-art CERT behavior-related dataset, producing groups with a density very close to the number of users.

## 1 Introduction

The spread of knowledge and technology across borders has intensified because of globalization. Transferring technology has boosted innovation and productivity. However, this favors the occurrence of sophisticated cyber attacks implemented by exploiting this technological evolution. This leads to numerous cybersecurity issues across cyberspace. Typically, cyber warfare refers to a series of malicious actions designed to disrupt critical infrastructures of a targeted country (Robinson et al., 2015). Nowadays, geopolitical issues contribute to the high prevalence of such a phenomenon (Serpanos and Komninos, 2022). Consequently, many organizations have improved their research on effective solutions to employ adequate countermeasures against the threat of cyber warfare. These solutions often refer to an interaction between skilled individuals and artificial intelligence (AI)-based techniques (Maher, 2017; Zhang et al., 2022; Potula et al., 2023). To centralize the benefits of combining different lines of defense, companies are increasingly equipped with a Security Operations Center (SOC) (Vielberth et al., 2020; Mughal, 2022). In the SOC context, the Security Information and Event Management (SIEM) is a central-role application (Cinque et al., 2018; Ban et al., 2023; Rosenberg et al., 2023). This tool is used to normalize security events from heterogeneous log sources and trigger alerts (Feng et al., 2017). It supports the efficiency of response to security incidents and provides an extended view of what was happening in the information technology (IT) ecosystem until the current moment (Podzins and Romanovs, 2019). The adoption of SIEM becomes paramount as IT organizations generate a large amount of data. This system fulfills the need to appropriately handle large data batches using sophisticated event correlation engines (Sekharan and Kandasamy, 2017). Recent studies have shown the effectiveness of SIEM in processing large volumes of data (Li and Yan, 2017). As a result, its employment has been investigated in several application domains, such as Internet of Things (IoT) networks (Zahid et al., 2023), smart city (Botello et al., 2020), Industrial Control System (ICS), and Operational Technology (OT) networks (Di Sarno et al., 2016; Fausto et al., 2021; Radoglou-Grammatikis et al., 2021).

Data analytics is a relevant feature of a SIEM platform. As a subfield of AI, machine learning (ML) involves data-driven algorithms that support the decision-making process of SOC analysts in detecting network intrusions (Anumol, 2015). In the current literature, several research works propose innovative AI-based intrusion detection methodologies (Das et al., 2019; Singh A. et al., 2022; Alkhudaydi et al., 2023; Maci et al., 2023, 2024; Park et al., 2023; Coscia et al., 2024). A SIEM can integrate these techniques to enhance real-time analysis capabilities (Muhammad et al., 2023). In this regard, the User and Entity Behavior Analytics (UEBA) engine aims to analyze the behavior of employees, third-party contractors, and collaborators of the organization to detect misbehavior in user activities (González-Granadillo et al., 2021). To achieve this purpose, UEBA typically employs ML algorithms trained on data collected from various sources, such as system logs, application logs, network devices, and network traffic (Khaliq et al., 2020). This engine represents a crucial component because legitimate users have greater privileged rights and authorized access to intranet resources than outsiders; therefore, these privileges can pose a potential high risk to the intranet if used in an unusual manner (Salitin and Zolait, 2018). Several investigations have addressed the problem of insider threat detection using ML algorithms, emphasizing the promising results obtained (Al-Mhiqani et al., 2020; Lavanya and Shankar Sriram, 2022; Bin Sarhan and Altwaijry, 2023). Modeling a user profile, including their interests, characteristics, preferences, and behaviors, is a crucial step in defining a suitable baseline to feed into ML algorithms that are capable of predicting deviations from them that are not known a priori (Eke et al., 2019; Savenkov and Ivutin, 2020). Unsupervised learning algorithms are useful when there is no prior knowledge of the anomaly being investigated (Vikram and Mohana, 2020; Carrera et al., 2022; Mochurad et al., 2023). A UEBA engine can greatly benefit from unsupervised learning algorithms because any substantial deviation from normal behavior in common communication patterns can represent a potential attack (Martín et al., 2021; Fysarakis et al., 2023). Clustering algorithms belong to the unsupervised learning paradigm and can be used in UEBA for different purposes, including anomaly detection, pattern recognition, and segmentation of user or entity behavior. Despite the crucial role that these algorithms could play in grouping similar entities based on behavioral patterns, selecting the most appropriate clustering algorithm for UEBA remains a challenging task due to the diverse nature of behavioral data and the need for real-time analysis, which in turn depends on the computational overhead of the method. The use of traditional clustering algorithms for UEBA was recently investigated by Datta et al. (2021) for real-time detection. However, while the recent literature provides some clustering applications for UEBA, these refer to traditional algorithms, i.e., no modern techniques are examined. Additionally, no multiple state-of-the-art datasets are taken into account for the experimentation, despite this aspect is essential to provide an extensive overview of the efficiency and robustness, i.e., the overall reliability of these algorithms in scenarios that are as close to reality as possible.

To address this challenge, we conduct a comprehensive investigation of multiple clustering algorithms and evaluate their applicability for UEBA based on several key aspects, such as scalability and performance effectiveness, in terms of the main properties of the derived groups, such as cohesion, separation, and density. Our thorough examination encompasses a wide range of clustering techniques, including traditional and more modern advances in such an algorithmic category. Furthermore, we provide a deeper understanding of the practical considerations and implementation aspects of these algorithms for UEBA, considering factors such as data pre-processing, feature selection, and algorithm parameter tuning. Such an analysis has the main objective of providing practical recommendations and advice in the complex process of determining the reliability of the evaluated methods to identify the most appropriate clustering algorithm for specific UEBA applications. The contributions proposed in this study can be summarized as follows:

- It represents a comprehensive review of clustering algorithms for UEBA, including the following key aspects at the data and algorithm levels:
  - Data-level: The investigation uses three distinct user behavior-related datasets, one of which consists of logs that represent real user actions, which is used for the first time in this kind of work. Each dataset undergoes tailored pre-processing strategies with the objective of making the data capable of preserving the scenario modeled, while reducing the computational effort required by the algorithms.
  - Algorithm-level: The investigation analyzes traditional and modern clustering methodologies, with an emphasis on their relevance to the UEBA domain, including a particular focus on scalability to address the big data aspect of the problem addressed. Each clustering technique undergoes a hyper-parameter tuning process, involving tailored metrics, to determine the most appropriate algorithm setting for specific UEBA scenarios.

The remainder of this study is organized as follows. Section 2 provides a review of the literature on approaches that address the UEBA challenge. The theoretical framework related to the algorithms evaluated in this study is described in Section 3. The experimental plan is presented in Section 4. In Section 5, the results of the proposed comparative study are listed. A critical evaluation of the results with the following main findings and insights is outlined in Section 6. The reliability analysis, which describes how clustering algorithms deal with UEBA, is provided in Section 7. Section 8 concludes this article and indicates possible future work.

## 2 Literature review

This section discusses the context in which our research occurs by describing advances in the literature that are related to our work and include: (i) general methodologies designed for UEBA; and (ii) clustering algorithms for UEBA. Finally, the proposed contribution motivation that emerged from such a revision is presented.

### 2.1 Methodologies for User and Entity Behavior Analytics

The deployment of increasingly sophisticated UEBA systems on real applications represents a fascinating challenge for the scientific community in several aspects (Dhillon et al., 2021). In Shashanka et al. (2016), a Single Value Decomposition (SVD)-based algorithm is implemented to create a behavior profile associated with a certain entity according to a baseline derived from a data preparation phase combined with a feature extraction strategy. Once the user behaviors are profiled, a confidence score is computed for each event by scoring the test features in relation to the behavior profile. Similarly, in the study by Yousef and Jazzar (2021), a SVD-based algorithm for UEBA is used to create a behavior profile per entity according to a group of entity-specific data processed appropriately. In Tang et al. (2017), user data are modeled using a factorization machine (FM) model, which is a recommender system-based method used to identify unusual access made by a user to any resource for the first time. This is achieved by learning from prior user-to-entity access logs and user context information subjected to an appropriate data preparation phase. In the study by Astakhova and Muravyov (2019), a UEBA solution is integrated with user behavioral biometrics and Open Source Intelligence (OSINT) technologies to increase the ability to recognize the operating context of the user. Specifically, according to the biometric recognition system, the generic user can be authorized or not to access a given resource. In the study by Yaochuang (2023), the presented UEBA engine combines a traffic analyzer with a system that examines the behavior of the Internet user. The latter uses a Generative Adversarial Network (GAN)-based model to determine whether a platform is legally accessed. In the study by Zunair Ahmed Khan et al. (2022), the UEBA problem is faced using time series analytical methods, such as the Scalable Time series Ordered-search Matrix Profile (STOMP) algorithm that creates a matrix profiling starting from a series of time-dependent user events. The methodology embeds the computation of the distance profile through a *z*-normalized Euclidean distance. The final matrix indicates the anomalous behavior based on the magnitude variation manifested by the user-related variables. In the study by Rashid and Miri (2021), UEBA engines are enriched using a differential privacy-based approach with the objective of preserving the privacy of the analyzed user data while retaining the accuracy of the anomaly detection model. This is achieved by perturbing the input data to the UEBA anomaly detection system by injecting Laplacian noise. In the study by Kaur et al. (2022), the UEBA challenge is addressed by examining the log data retrieved using the Cloud Trail Logs tool. Then, these data are pre-processed according to *ad hoc* data cleaning and feature engineering strategies to create a user behavior baseline. The latter is used to control whether new logs can be considered anomalous or not by computing a threat index that is a function of the deviation between the current log data and the baseline. A general framework for detecting insider threats from user behavior is provided in Singh M. et al. (2022). It consists of a combination of feature engineering techniques, such as isometric feature mapping and content-based feature extraction, to minimize information loss while providing a set of optimal features to a multi-fuzzy classifier via an emperor penguin feature selection algorithm. UEBA engines are correlated with data analysis and visualization tools to identify user misbehavior, even in the cases of multiple organization scenarios, such as in the study by Rengarajan and Babu (2021). In the study by Piñón-Blanco et al. (2023), an UEBA application in OT networks is used characterize the interactions between operators and ICSs, defining the entities of interest to create a baseline. Then, such a baseline is transmitted to a deep learning (DL)-based anomaly detection system that employs a Long Short-Term Memory (LSTM) model. Similarly, a LSTM model is leveraged in the study by Sharma et al. (2020) as an autoencoder to learn the user behavioral pattern (known in advance, i.e., such method works on labeled data) and to determine anomalies according to a threshold. The latter is computed on the basis of a reconstruction error defined on the legitimate data subset. DL models are also used in the study by Singh et al. (2019) for user profiling. Specifically, this method consists of a combination of a multi-state LSTM and a convolutional neural network (CNN) that is capable of learning behavioral patterns over time. Using UEBA engines, the security of classical applications, such as the Federated Identity Management (FIM), can be improved (Martín et al., 2021). In particular, a session fingerprint is defined to characterize the user (within the identity federations) behavior and misbehavior. In the study by Najafi et al. (2021), a natural language processing (NLP)-based approach is presented to discover anomalies in entity behavior. In such a study, the entities considered were executable files, and the presence of outliers in their behaviors could be considered a possible symptom of the presence of malicious executable software. In the study by Lukashin et al. (2020), a scalable data processing framework is proposed to address the UEBA problem. It comprises a set of modules that are delegated to extract the most relevant data parameters according to a particular topic and to transform them for ML-based anomaly detection tools, such as LSTM, Isolation Forest, and Support Vector Machine (SVM). Recent investigations have highlighted the widespread use of several clustering algorithm configurations for UEBA, denoting the need to examine in this direction (Landauer et al., 2020; Sarker, 2021).

### 2.2 Clustering algorithms for User and Entity Behavior Analytics

In the study by Iglesias Perez and Criado (2023), *K*-means algorithm applied to time series data is used in combination with graph analysis techniques on information extracted from an UEBA engine realized through temporal behavior multiplex networks. In the study by Parwez et al. (2017), *K*-means and hierarchical clustering algorithms are employed to detect anomalies in user communication patterns in the context of mobile networks. Specifically, anomalies are determined according to the number of samples that fall into the generic inferred cluster. Less-numerous clusters are considered anomalous. In the study by Hu et al. (2018), the presented UEBA engine consists of four components: a data log collector, data log analyzer, behavior profile storage, and anomaly detector. In such a setting, the analyzer module uses the agglomerative clustering algorithm to establish the baseline according to a custom similarity metric. The anomaly detection module identifies unusual behavior in user actions by comparing them with a predefined baseline, triggering an anomaly alert when the deviation exceeds a set threshold. Similarly, in the study by Kim et al. (2019), a *K*-means-based misbehavior detection algorithm is investigated. Specifically, training samples are used to define a baseline used to categorize anomalous or not, never-seen-before samples. This is achieved by introducing an anomaly score, given by the ratio between the distance from the closest centroid and that relative to the centroid itself. In the study by Ariyaluran Habeeb et al. (2019), the authors proposed a clustering-based methodology and compared its performance with that of several algorithms, including *K*-means, spectral clustering, agglomerative clustering, and Hierarchical Density-Based Spatial Clustering of Applications with Noise (HDBSCAN) for real-time anomaly detection in entity behaviors within a IoT context. In the study by Wang et al. (2016), user behavior models were extracted by applying hierarchical cluster methodologies to a graph data structure. Modern recommender systems are trained to predict the potential future interactions made by users with entities on the basis of data collected about the past behavior of users. In the study by Xie and Wang (2018), a two-stage clustering algorithm was used to provide recommendations. The first stage uses a density-based clustering method to produce the number of clusters to be used as input to the second phase, which employs *K*-means. Given the widespread use of the *K*-means algorithm, several versions of this method have been proposed in the current literature (Ikotun et al., 2023). Each variant updates a specific mechanism with respect to the vanilla operating mode to gain the desired advantage, such as the scalability of the algorithm when dealing with big data. For instance, the mini-batch *K*-means considers a reduced number of training samples to perform the center positioning update. The nested mini-batch *K*-means method uses repurposed mini-batches in consecutive training iterations. Alternatively, *K*-means++ restructures the centroids' initialization strategy using a specific selection probability. The latter strategy has been proposed in behavior-related data grouping applications. Specifically, in the study by Mayhew et al. (2015), *K*-means++ is used to cluster different categories of machines, such as servers or desktops, as well as web servers or web crawlers. In the study by Gao et al. (2019), the analysis of user behavior clusters is performed by comparing the results obtained using both *K*-means and *K*-means++. As an alternative to partitioning-based *K*-means, fuzzy c-means searches for clusters according to the computation of a data structure (a matrix) that defines the probability that a sample belongs to a certain group. This algorithm was used in the study by Castellano et al. (2007) for user profiling purposes. In the study by Cui et al. (2022), a fuzzy clustering algorithm is improved by using a particle swarm optimization procedure that refines the research on centroid categories. Such an UEBA approach helps solve the problem of fuzzy clustering results that easily converge to the local optimum. Furthermore, fuzzy membership matrices are used to express the probability of being an anomaly. In the study by Zola et al. (2021), two state-of-the-art density-based clustering algorithms, namely, Density-Based Spatial Clustering of Applications with Noise (DBSCAN) and Ordering Points to Identify the Clustering Structure (OPTICS), are compared with HDBSCAN in the task of grouping the behavior of normal entities according to their similarity. As part of a detection framework that performs a behavioral analysis of online users, OPTICS is used to infer the relationship between user-related events on social networks (Nguyen and Jung, 2017). Various purposes can be achieved with user behavior pattern analysis, such as identifying the location of the user as in the study by Madhur Arora and Patel (2023). Specifically, the Balanced Iterative Reducing and Clustering using Hierarchies (BIRCH) algorithm is applied to cluster the trajectory data referred to the position coordinates of the user. In the study by Kuiper et al. (2019), a framework is presented that leverages a combination of hierarchical and partitioning clustering algorithms to profile user behaviors. In particular, hiearchical clustering is used to determine the number of clusters to configure the next non-hierarchical procedure. The effectiveness of hierarchical clustering algorithms for time-series-based tasks, such as UEBA, is highlighted in the study by Meng et al. (2023). However, the authors of both articles observed that despite their effectiveness, a major drawback regards their inability to handle large datasets. To overcome such limitations, recent advances have been made in the proposal of scalable and accurate hierarchical clustering algorithms such as First Integer Neighbor Clustering Hierarchy (FINCH) (Sarfraz et al., 2019) and Sub-Cluster Component (SCC) (Monath et al., 2021). Other recent directions explored in the sense of scalable clustering procedures relied on the use of subspace-based techniques, so that the total space dimension is reduced to apply clustering in a lightweight mode while retaining an high accuracy. Traditional applications have already been investigated for anomaly detection tasks, such as insider threat detection (Pichara and Soto, 2011). In recent years, subspace clustering has been applied considering the ensembles of several (as many as searched groups) (Lipor et al., 2020), mainly because of the introduction of effective approaches such as Sparse Subspace Clustering by Orthogonal Matching Pursuit (SSC-OMP) (You et al., 2016b) and Elastic net Subspace Clustering (EnSC) (You et al., 2016a) methodologies. Kan et al. (2023) presented a model based on a set of statistical techniques and Guassian Mixture Models (GMMs) to identify user-level malicious behaviors, defined as deviations from a nominal baseline. Density-peak clustering methods, such as Density peak-based clustering using Mutual nearest neighbors (DenMune) (Abbas et al., 2021), have recently been applied to the UEBA task (Liu, 2022). This algorithm class is mainly based on the construction of a decision graph according to distance and density measures. It is possible to choose the cluster centers explicitly on the decision graph based on the decision graph. Other data points can then be grouped into clusters based on their closest and densest neighbors once the cluster center has been determined. In the study by Datta et al. (2021), a comparison between four algorithms is presented, namely *K*-means, agglomerative, DBSCAN, and GMM. This evaluation was conducted using a single dataset that modeled the interaction between users and e-commerce platforms. Algorithm performance was recorded using external validation metrics that show the effectiveness of *K*-means and agglomerative clustering algorithms.

### 2.3 Motivation

From the above literature review section, the lack of a thorough evaluation of clustering algorithms has emerged. Moreover, existing (not comprehensive) analyses have been limited to the validation of quantitative performance, without providing any interpretation of the groups generated by the algorithms. A broader analysis would consider a larger number of datasets and investigate the impact of the hyper-parameter setting on the performance of such methodologies. In addition, the reliability of recent state-of-the-art clustering algorithms has not yet been examined when dealing with the UEBA case study. In light of this, we provide a comprehensive review of clustering algorithms for UEBA with the aim of pointing out the advantages and disadvantages of 15 clustering algorithms, from classical to recent advances, selected according to their application in UEBA-related tasks as discussed in the aforementioned section. Furthermore, to the best of our knowledge, there is no evidence of the application of scalable clustering algorithms to this problem. As a consequence, our review is the first study that considers some clustering algorithms, such as SSC-OMP and EnSC that has already showed their suitability for big data challenges similar to UEBA. Finally, to fill the highlighted gaps, our investigation considers three state-of-the-art UEBA datasets and evaluates the internal performance achieved by each clustering algorithm for different hyper-parameter configurations. Taking into account the best configuration emerging from the fine-tuning phase, an analysis of the density of clusters generated by the algorithms was performed to validate the internal performance of the methods based on the number of samples within each generated cluster.

## 3 Clustering algorithms selected for this investigation

This section lists the clustering algorithms used in this article to face the UEBA challenge, an example of an industrial application that, as discussed so far, fits well with these algorithms (Benabdellah et al., 2019). As a general rule, this type of algorithm can be divided into partitioning and hierarchical methods. In the first case, a predetermined number of distinct groups are created by systematically partitioning the input data *X*, which are composed of *m* samples, each with *n* features. Depending on the techniques used in creating the clusters and the nature of the clusters generated, partitioning clustering algorithms can be classified as hard/crisp (density-based, subspace, etc.), fuzzy, and mixture resolving. In the second case, data are hierarchically arranged into a series of layers, so clusters are built on top-down (divisive approach) or bottom-up (agglomerative approach) dendrograms (Ezugwu et al., 2022). Algorithms belonging to both categories are considered in this study. Furthermore, we examine scalable clustering methods. This category of techniques can handle extremely large data by scaling the execution to tackle the clustering computational overhead of big data. These algorithms can be categorized according to the chosen scaling methodology. This study considers the following scalable clustering methods (Mahdi et al., 2021): (i) sample-based, which infers about a subset of data, generalizing the results obtained over the full dataset; and (ii) subspace, which performs cluster searching in a vector space reduced with respect to the starting one. Table 1 summarizes the categorization of the clustering algorithms used in this investigation.

**Table 1:** Categorization of clustering algorithms applied in this study.

**Algorithm**	**Category**			
	**Partitioning**	**Hierarchical**	**Scalable**	
			**Sample-based**	**Subspace**
*K*-means	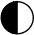	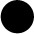	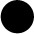	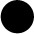
Fuzzy c-means	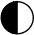	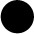	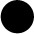	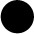
GMM	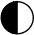	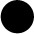	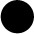	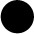
DBSCAN	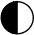	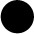	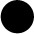	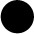
BIRCH	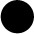	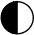	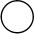	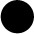
OPTICS	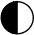	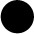	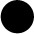	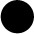
Mini-Batch *K*-means	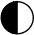	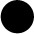	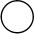	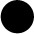
Scalable *K*-means++	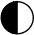	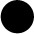	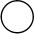	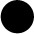
HDBSCAN	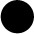	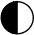	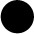	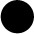
Nested mini-batch *K*-means	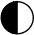	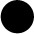	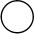	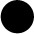
SSC-OMP	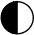	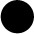	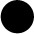	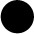
EnSC	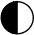	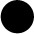	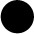	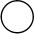
FINCH	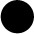	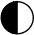	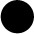	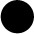
SCC	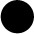	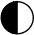	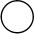	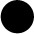
DenMune	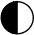	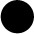	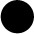	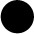

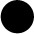
 : Algorithm does not fall into the category.

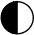
 : Algorithm falls into partitioning or hierarchical category.

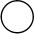
 : Scalable partitioning/hierarchical clustering algorithm.

### 3.1 Partitioning

#### 3.1.1 *K*-means

The *K*-means (MacQueen et al., 1967) represents the most classic partitioning-based clustering algorithm. It requires selecting the number of clusters (usually indicated with *K*) to search for. Then, *K* centroids are initialized and assigned to each cluster. Given a data point *x*_*i*_∈*X*, with *i* = 1, ..., *m*, each algorithm iteration attempts to fit such a point into the most suitable cluster by comparing it with the *k*−*th* cluster centroid, where *k* = 1, ..., *K*. Specifically, the centroid closest (in terms of a certain distance metric, e.g., the Euclidean distance) to *x*_*i*_ is selected. The *K*-means algorithm continues by adjusting the positioning of each centroid (i.e., updating the centroid set C, with |C|=K), which is defined for each iteration according to the minimization of a cost function (Ezugwu et al., 2022):


(1) $$\begin{array}{l} \zeta \left(C\right)=\overset{K}{\underset{k=1}{\sum }}\overset{m}{\underset{i=1}{\sum }}\lambda_{ik}∥x_{i}-\mu_{k}{∥}^{d} \end{array}$$

where μ_*k*_ is the mean value of the data points assigned to cluster *k* (that corresponds to the center of the *k*-th cluster), while λ_*ik*_ = 1, if *x*_*i*_ belongs to the *k*−*th* cluster; otherwise, it is null. Finally, *d* specifies the type of distance function (*d* = 2 for the Euclidean distance). Each point in the dataset is eventually assigned a different group based on the displacement of the centroids until a stopping condition is met, e.g., the end of training iterations *T* is reached.

#### 3.1.2 Fuzzy c-means

The fuzzy c-means (Bezdek et al., 1984) is based on the production of *K* partitions for *X* by searching for similarity between *x*_*i*_∈*X* and the *k*-th cluster. This relationship is expressed using a membership value (∈[0, 1]), which is computed using a membership function. Unlike the *K*-means algorithm, the fuzzy partitioning process is achieved through an iterative optimization of the following function (which is slightly modified compared to Equation 1):


(2) $$\begin{array}{l} J_{r_{n}}\left(C\right)=\overset{K}{\underset{k=1}{\sum }}\overset{m}{\underset{i=1}{\sum }}u_{ik}^{r_{n}}∥x_{i}-c_{k}{∥}^{d} \end{array}$$

where in Equation (2): (i) *r*_*n*_∈ℝ, *r*_*n*_>1; (ii) *u*_*ik*_ represents the degree of membership of *x*_*i*_ with respect to the *k*-th cluster; (iii) *c*_*k*_ is the centroid of dimension *d*. This process results in the update of centroid placements and membership values that determine the so-called membership matrix *U*∈ℝ^*m*×*K*^ as follows:


(3) $$\begin{array}{l} u_{ik}=\frac{1}{\overset{K}{\underset{k=1}{\sum }}{\left(\frac{∥x_{i}-c_{z}∥}{∥x_{i}-c_{k}∥}\right)}^{\frac{2}{r_{n}-1}}} \end{array}$$

where $c_{z}=\frac{\overset{m}{\underset{z=1}{\sum }}u_{iz}^{r_{n}}x_{i}}{\overset{m}{\underset{z=1}{\sum }}u_{iz}^{r_{n}}}$. Training continues until no centroid adjustments are made in the clusters found.

#### 3.1.3 Gaussian mixture models

GMMs (Mclachlan and Basford, 1988) are used to represent sub-populations normally distributed within an entire population. Specifically, a point within a set of data can be assigned to a normal Gaussian density function characterized by specific mean and standard deviation values. However, if multiple Gaussian distributions that can represent this point exist, a mixture model consisting of the marginal aggregation of such distributions can be considered to indicate the probability of each distribution. A GMM can be formally expressed as:


(4) $$\begin{array}{l} p\left(x_{i}|\theta \right)=\overset{K}{\underset{k=1}{\sum }}\upsilon_{k}p_{k}\left(x|\theta_{k}\right) \end{array}$$

where: (i) υ_*k*_ is the probability of the *k*-th Gaussian among all distributions, also called the mixing coefficient; and (ii) *p*_*k*_(*x*) represents the density probability of the *k*-th Guassian given the point *x*_*i*_∈*X*. Recall that a multivariate (*n* dimension) Guassian under the mixture perspective can be defined as $p\left(x_{i},\mu_{k},\Sigma_{k}\right)=\frac{e^{-\frac{1}{2}{\left(x_{i}-\mu_{k}\right)}^{T}\Sigma_{k}^{-1}\left(x_{i}-\mu_{k}\right)}}{\sqrt{{\left(2\pi \right)}^{n}|\Sigma_{k}|}}$, with $\mu_{k}=\frac{1}{\left|C_{k}\right|}\underset{x_{i}\in C_{k}}{\sum }x_{i}$ the vector of the means of the *k*-th Gaussian, whereas $\Sigma_{k}=\frac{1}{|C_{k}|-1}\underset{x_{i}\in C_{k}}{\sum }\left(x_{i}-\mu_{k}\right){\left(x_{i}-\mu_{k}\right)}^{T}$ is the covariance matrix of the *k*-th Gaussian. In Equation (4), θ and θ_*k*_ represents the parameters of all Gaussian distributions and *k*-th, respectively. To find the most suitable parameterization, the so-called Expectation Maximization (EM) algorithm is used. It consists of two main phases. First, μ_*k*_, Σ_*k*_, and υ_*k*_ are randomly set for all *k*∈*K*. Subsequently, for each pair of cluster points, the responsibility coefficient (see Equation 5, where *t* corresponds to the training step) is calculated during the expectation step to quantify the extent to which the point is generated from the currently paired Gaussian in relation to the entire mixture. Then, it is used to recompute all the aforementioned parameters to maximize the likelihood until a convergence criterion is met.


(5) $$\begin{array}{l} r_{ik}=\frac{\upsilon_{k}p_{k}\left(x_{i}|\theta_{k}^{\left(t-1\right)}\right)}{\overset{K}{\underset{k=1}{\sum }}\upsilon_{k}p_{k}\left(x_{i}|\theta_{k}^{\left(t-1\right)}\right)} \end{array}$$

#### 3.1.4 DBSCAN

Density-based clustering algorithms fall into the partitioning category (Ezugwu et al., 2022). The DBSCAN (Ester et al., 1996) represents one of the most popular algorithms that belong to this class. The concept of density defines the distribution of subsets of points in *X*. In particular, a high distribution of points is said to be dense and more representative of the presence of a cluster. Furthermore, the density of the noise areas is typically lower than that of the clustered points. To apply such logic in the desired vector space, each point *p* belonging to a cluster must be correlated with the so-called ε-neighborhood $N{\left(p\right)}_{\varepsilon }=\left\{q\in X|dist\left(p,q\right)\leq \varepsilon \right\}$. Therefore, the latter is characterized by a radius defined according to a distance metric calculated with respect to a point *q*, resulting in a suitable density (a minimum number of points *m*_*pts*_∈ℕ is part of the neighborhood). *q*_*c*_ is said to be a core point if $|N{\left(q_{c}\right)}_{\varepsilon }|>m_{pts}$. These are the points located in the interior of a cluster. On the other hand, *q*_*b*_ is denoted as a border point if $|N{\left(q_{b}\right)}_{\varepsilon }|<m_{pts}$ and $q_{b}\in N{\left(q_{c}\right)}_{\varepsilon }$. Points that are neither cores nor borders are called noise points. Whenever two core points are close enough (within ε) to each other, they are grouped together. Border points close to the core points are placed in the same cluster. Finally, the noise points are discarded. This is achieved through the so-called reachability and connectivity relationships:

- Point *q* is density-reachable from point *p* if $q\in N{\left(p\right)}_{\varepsilon }$ and *p* is a core point. This relationship is symmetric when both points are core points but is not symmetric when a border point is part of the pair.
- Point *p* is density-connected to point *q* if there is another point *r* such that both *p* and *q* are density-reachable from *r*. This relation is symmetric.

To summarize the execution of DBSCAN, it starts searching for all points density-reachable from *x*_*i*_∈*X* and initializes a cluster if *x*_*i*_ is a core point. Given the list of core points found, the algorithm retrieves all connected points to a single core point and joins the clusters. Finally, all points in *X* deprived of core points are noise if their distance from the closest cluster is greater than ε.

#### 3.1.5 OPTICS

The OPTICS (Ankerst et al., 1999) is a density-based algorithm that extends DBSCAN. This variation relies on the idea of simultaneously building density-based groups for different and theoretically infinite ε′ values, such that 0 < ε′ < ε. This is achieved by taking advantage of two main concepts: (i) core-distance; and (ii) reachability-distance. For a point *p*∈*X*, the core-distance is defined as the smallest distance from *p* to another point within its ε-neighborhood, specifically, the distance between *p* and its *m*_*pts*_-th nearest neighbor. On the other hand, the reachability-distance of *p* in relation to another point *q* is the smallest distance such that *p* is directly density-reachable from *q*, provided that *q* is a core object. These two measures are used to sort points in *X* to extract all density-based clusters for different ε′ < ε values. After the order of points is obtained, groups of data are defined as such. In this study, to improve the OPTICS performance, the version presented in the study by Kamil and Al-Mamory (2023) is considered. Specifically, it comprises two phases. The fuzzy c-means algorithm is applied to the original dataset to obtain the membership matrix (see Equation 3), which represents the data used by OPTICS for training. In this way, because fuzzy c-means already provide a cluster map, the search space of the OPTICS algorithm is reduced; in fact, the algorithm will consider the neighbors within a single cluster of points, ignoring all the rest.

#### 3.1.6 Mini-batch *K*-means

The mini-batch variant of the *K*-means (Sculley, 2010) addresses the scalability of the original algorithm at the optimization level. In particular, in the original algorithm, Equation (1) is minimized using a gradient descent algorithm that typically converges to a local optimal solution iterating throughout the entire dataset. In the case of mini-batch *K*-means, the dataset is split into small subsets, namely mini-batches $M_{b}$ ($|M_{b}|<m$), and the gradients are computed for each of them. Such an approach provides a trade-off between the original implementation and that that uses a stochastic-based gradient descent optimization procedure, thereby gaining robustness and computational efficiency.

#### 3.1.7 Scalable *K*-means++

The scalable *K*-means++ (Bahmani et al., 2012) extends the centroid initialization methodology proposed in the study by Arthur and Vassilvitskii (2007), improving scalability by reducing the number of steps required to effectively set up parallel computation. Specifically, it consists of updating the initial *K*−1 centroids (the first is a point sampled uniformly from *X*) according to a non-uniform probability function $l\frac{∥x_{i}-c_{i}{∥}^{d}}{\zeta }$, where *x*_*i*_∈*X* and $c_{i}\in C$, while *l* = Ω(*K*) is an oversampling factor. Because this strategy results in an initial configuration of C such that |C|>K, each point in the so-far formed C is weighted considering the number of points in *X* closer to it than any other point in C. Then, these computed weights are used to perform new clustering in such a way that the condition |C|=K holds.

#### 3.1.8 Nested mini-batch *K*-means

The nested mini-batch *K*-means (Newling and Fleuret, 2016) constitutes an extension of the mini-batch version of *K*-means in the sense of the adopted mini-batch sampling strategy. Specifically, instead of randomly selecting equal-sized mini-batches, they are selected in a nested manner. This is achieved by satisfying the constraint $\left|M_{b_{t}}|\leq |M_{b_{t+1}}\right|$, with *t*∈{1, ..., *T*−1} being the current training iteration. In this criterion, it is paramount to properly select the size of the mini-batch for each *t*. The motivation behind this choice is better understood by examining two different hypothetical size updates examined between consecutive steps: (i) $|M_{b_{t+1}}|=|M_{b_{t}}|=S_{1}$; (ii) $|M_{b_{t+1}}|=2\times |M_{b_{t}}|=S_{2}$. Denoting by *c*_*k*_*t*__ the *k*-th centroid and $c_{k_{t+1}}\left(\left|M_{b_{t+1}}\right|\right)$ and the *k*-th centroid given a mini-batch of size $\left|M_{b_{t+1}}\right|$ at steps *t* and *t*+1, respectively. To avoid redundancy in the centroid update and a much larger change in the centroid (premature fine-tuning condition), the estimation presented in Equation 6 is proposed:


(6) $$\begin{array}{l} ||c_{k_{t+1}}\left(S_{2}\right)-c_{k_{t+1}}\left(S_{1}\right)||\to \frac{1}{2}\sqrt{\frac{1}{|M_{b_{t}}{|}^{2}}\overset{\left|M_{b_{t}}\right|}{\underset{i=1}{\sum }}||x_{i}-c_{k_{t}}{||}^{2}}=\frac{1}{2}{\hat{\sigma }}_{C}{\left(k\right)}^{2} \end{array}$$

Given the initial size of the first mini-batch, the nested *K*-means algorithm iterates until $\underset{k}{\min}\frac{{\hat{\sigma }}_{C}\left(k\right)}{∥c_{k_{t}}-c_{k_{t+1}}\left(\left|M_{t}\right|\right)∥}$ is higher than a threshold that reflects the relative costs of premature fine-tuning and redundancy.

#### 3.1.9 SSC-OMP

The SSC-OMP algorithm (You et al., 2016b) exploits the self-expressiveness property of the data so that each point in a union of subspaces can be expressed as a linear combination of other points in the subspaces, i.e., *X* = *XC*, with *diag*(*C*) = 0. Of all possible coefficient matrices *C*, the so-called subspace-preserving is searched. Such a matrix enables the representation of a point in a subspace as a linear combination of η alternative points in the same subspace. Once found, the affinity matrix *W* can be obtained (so that given two points *x*_*i*_ and *x*_*j*_, *w*_*ij*_ = |*c*_*ij*_|+|*c*_*ji*_|) to group the data using spectral clustering algorithms. The main objective of the SSC-OMP algorithm is to find a sparse representation of each point in terms of other data points. This is achieved by solving the following optimization problem:


(7) $$\begin{array}{l} c_{i}^{*}=\underset{c_{i}}{arg min}∥x_{i}-Xc_{i}{∥}_{2}^{2}\text{s.t.}∥c_{i}{∥}_{0}\leq k \end{array}$$

The objective function in Equation (7) is minimized using the orthogonal matching pursuit algorithm, which computes the subspace-preserving matrix C^*^ to derive *W* = |C^*^|+|C^*T^|. Finally, the segmentation of *X* is obtained by clustering the affinity matrix using the spectral clustering algorithm used in the vanilla sparse subspace clustering method (Elhamifar and Vidal, 2009).

#### 3.1.10 EnSC

The EnSC (You et al., 2016a) represents an alternative geometric method compared to the methods based on sparse subspaces in the mechanism used to calculate *C*. This is achieved by referring to the following objective function:


(8) $$\begin{array}{l} \underset{c_{i}}{\min}\lambda ∥c_{i}{∥}_{1}+\frac{1-\lambda }{2}∥c_{i}{∥}_{2}^{2}+\frac{\lambda }{2}∥x_{i}-Xc_{i}{∥}_{2}^{2}\text{s.t.}c_{ii}=0 \end{array}$$

where λ∈[0, 1). From Equation (8), $\delta_{i}=\lambda \left(x_{i}-Xc_{i}^{*}\right)$ is called the oracle point and cannot be found until $c_{i}^{*}$ is found. Given such a point, the oracle region is derived. It consists of two opposite spheres ℝ^*n*^ symmetrically located at $\pm \frac{\delta }{∥\delta {∥}_{2}}$. To compute the oracle region, the so-called ORacle Guided Elastic Net (ORGEN) method is used, which addresses such a problem by sequentially solving reduced-scale subproblems. First, the active (support) set T is initialized (authors advise λ = 0; note that such a parameter defines the size of the oracle region, so that to small values corresponds a large oracle region and well-connected points) and is used to compute the oracle region. Then, T is updated considering the actual support vectors. The procedure is repeated for a finite number of iterations. In such a setting, the subspace-preserving condition is reached if the following inequality $\frac{\lambda }{∥\delta \left(x_{i},X_{i}^{l}\right){∥}_{2}}\geq \frac{r_{i}^{2}}{r_{i}+\frac{1-\lambda }{\lambda }}$ is met, where *x*_*i*_ is a point in a subspace with dimension *l*<*n* and *r*_*i*_ is the inradius of the convex hull of the symmetrized points in $X_{i}^{l}$, that is, a dictionary contained in the subspace itself.

#### 3.1.11 DenMune

The DenMune (Abbas et al., 2021) takes advantage of the principle of consistency of *K*_*nn*_-mutual-nearest-neighbors such that pints that share some mutual nearest neighbors must be part of the same cluster. In this case, *K*_*nn*_ is a user parameter that defines the size of a neighbor. Consider *x*_*i*_∈*X*, the *K*_*nn*_-nearest neighbors of such a point can be interpreted as a list of sorted points according to their distance from a reference point denoted by *KNN*_*x*_*i*_→_. On the other hand, if *x*_*i*_∈*KNN*_*x*_*j*_→_, with *x*_*j*_∈*X*, the following condition *x*_*j*_∈*KNN*_*x*_*i*_←_, holds. The set of mutual nearest neighbors of *x*_*i*_ is defined as the intersection between *KNN*_*x*_*i*_→_ and *KNN*_*x*_*i*_←_. In essence, this set identifies dense points with respect to *x*_*i*_. To better isolate dense points, the DenMune algorithm implements a scoring mechanism to classify the data samples into: (i) strong points if $\frac{\left|KNN_{x\leftarrow }\right|}{K_{nn}}\geq 1$; (ii) weak points if $\frac{\left|KNN_{x\leftarrow }\right|}{K_{nn}}<1$; and (iii) noise points if $0\leq \frac{\left|KNN_{x\leftarrow }\right|}{K_{nn}}<<1$. Strong points are used to construct clusters, then some of the appropriate weak points (those with $\frac{\left|KNN_{x\leftarrow }\right|}{K_{nn}}$ close to 1) are merged with the existing clusters, and the remaining part with the noise points is discarded.

### 3.2 Hierarchical

#### 3.2.1 BIRCH

The BIRCH (Zhang et al., 1996) is a classical hierarchical algorithm used to handle large datasets. Clustering feature (CF) tuples are used to summarize information in dense regions or clusters of data to minimize the memory requirements of large datasets. This tuple is expressed as *c*_*f*_ = < *m, f*(*m*), *g*(*m*)>, where *f* and *g* perform linear and squared summations of the data points, respectively. According to this formulation, the so-called CF tree can be used to compactly represent subclusters within *X* as a leaf node. CF trees contain CF entries that are made up of the sum of the CF entries in their children. This is achieved according to the CF additive theorem, which states that given *c*_*f*_1__ = < *m*_1_, *f*_1_(*m*_1_), *g*_1_(*m*_1_)> and *c*_*f*_2__ = < *m*_2_, *f*_2_(*m*_2_), *g*_2_(*m*_2_)>, *c*_*f*_1__+*c*_*f*_2__ = < *m*_1_+*m*_2_, *f*_1_(*m*_1_)+*f*_2_(*m*_2_), *g*_1_(*m*_1_)+*g*_2_(*m*_2_)>. The maximum number of entries in each leaf node is set according to a threshold, which also influences the tree size. Once the CF tree is built, a global clustering phase is performed that can employ the above-cited clustering algorithms, such as *K*-means.

#### 3.2.2 HDBSCAN

The HDBSCAN (Campello et al., 2013) presents a hierarchical-based variant of the previously described DBSCAN algorithm. In this approach, density estimation takes advantage of the concept of core distance. Given a point *p*∈*X*, *d*_*core*_(*p*) indicates the distance from *p* to the *m*_*pts*_-nearest neighbor. The core distances of the points in the denser regions are generally smaller, whereas those in the sparser regions are larger. This metric is then used to define the mutual reachability distance between *p, q*∈*X*, as *d*_*mreach*_(*p, q*) = *max*{*d*_*core*_(*p*), *d*_*core*_(*q*), *dist*(*p, q*)}. This measure is used to build the mutual reachability graph, where each node is a generic data point, and the generic edge that connects two nodes is weighted by the mutual reachability distance of the involved nodes. If such a graph is pruned of arcs having a weight higher than ε, it makes sense that clusters are generated by the connected components of the ε-core points, i.e., objects with *d*_*core*_(*p*) ≤ ε. The remaining points are assumed to be noise. Such a drop operation can be performed by adopting methods capable of searching for the minimum spanning tree of the graph. Then, the minimal spanning tree is transformed into a hierarchy of connected components that need to be clustered according to a minimum cluster size (*m*_*cluste*_*r*__*size*__), i.e., a value above which a set of points is considered as a cluster. Among the clusters thus identified, only those defined as stable are retained, according to a persistence criterion defined by analyzing the inverse distance values when the generic cluster is generated.

#### 3.2.3 FINCH

The FINCH (Sarfraz et al., 2019) algorithm is a parameter-free hierarchical clustering procedure. Given *x*_*i*(*j*)_∈*X*, the affinity matrix *A* can be calculated as follows:


(9) $$A(x_{i},x_{j})=\{\begin{array}{ll} 1 & \text{if }x_{j}=\kappa_{x_{i}}^{1}\text{or }x_{i}=\kappa_{x_{j}}^{1}\text{or }\kappa_{x_{i}}^{1}=\kappa_{x_{j}}^{1}\\ 0 & \text{otherwise} \end{array}$$

where $\kappa_{x_{i\left(j\right)}}^{1}$ is the first neighbor of point *x*_*i*(*j*)_. The matrix obtained from Equation 9 directly infers the grouping relationship between data points that must be recursively merged. This is achieved by averaging the data samples within each group and using the mean vectors to determine the first nearest neighbor. As specified above, the FINCH algorithm comes as a free parameter procedure. However, the overall algorithm can be adapted to return the desired number of clusters *K* (if required) by refining a closed partition in the returned hierarchy tree.

#### 3.2.4 SCC

The SCC (Monath et al., 2021) determines which points should belong to the same cluster in a sequence of rounds (the single round is indicated with τ). First, SCC places each point into its own distinct cluster. Then, a merging operation is applied to the cluster sets if the sub-cluster component criterion is fulfilled. Taking into account a set of data *X*, it can be flat in a set *S* = {*m*_1_, *m*_2_, ..., *m*_*K*_}, i.e., $\cup_{k=1}^{K}m_{k}=X$. Two sub-clusters *m*_*l*_, *m*_*z*_∈*S* belong to the same sub-cluster component [*m*_*H*_*f*__(*m*_*l*_, *m*_*z*_, ρ, *S*) = 1] according to a threshold ψ and a linkage function $f:P\left(X\right)\times P\left(X\right)\to {\mathbb{R}}^{+}$, with P(X) the set of all possible *S*, if there exists a path from *m*_*l*_ to *m*_*z*_ in a hierarchical arrangement such that (i) *f*(*m*_*s*_*r*__, *m*_*s*_*r*−1__) ≤ ρ for 0 ≤ *r* ≤ *z*; and (ii) *m*_*s*_*r*−1__ = argmin_*m*∈*S*_*f*(*m*_*s*_*r*__, *m*)∧(∨)*m*_*s*_*r*__ = argmin_*m*∈*S*_*f*(*m*_*s*_*r*−1__, *m*). Therefore, in a round τ_*t*_, the inference is produced considering the sub-clusters merged in the previous round that are in the same sub-cluster component. Note that when no clusters are merged in the previous round, ψ increases. In essence, sub-cluster components can be conceptualized as the connected components of a graph, with sub-clusters (nodes) from the previous round and arcs connecting pairs of nodes with a distance less than ψ between them.

## 4 Experimental setup

First, this section describes the datasets used. For each, a specific pre-processing strategy has been adopted and detailed. Second, the metrics used to evaluate the selected clustering algorithms are described and discussed. Third, the implementation details and algorithm settings are listed.

### 4.1 User and Entity Behavior Analytics Data

#### 4.1.1 Description

To provide an extensive comparative study, three different problem-specific datasets were selected according to their usage in the current literature. To simplify the notation, the datasets are identified by integers. Their description is as follows:

- The first dataset (D1) used is called E-shop Clothing (Łapczyński and Białowa̧s, 2013, 2019). This has been selected because it was used in the study by Datta et al. (2021) to evaluate a set of four clustering algorithms for the same problem addressed in this study. D1 is a collection of information on the clickstream of an online store that can be used to identify behavioral patterns starting from the interaction between users and the web application. It consists of the study by Diwandari and Zaky (2021):
  1. Timing data are divided into three different variables, namely year, month, and day, each encoded as an integer number (e.g., 29*th* April 2008 is expressed as year=2008, month=4, and day=29).
  2. Geographic data are represented by a single feature, namely country, in which the country of origin of the IP address is indicated by a numerical label (e.g., country=1 to indicate Australia). Any label is encoded by the authors using their own dictionary.
  3. Session and web page data collection (all integer numbers), including: (i) session ID; (ii) page 1 (main category) that concerns the main category to which the product belongs, based on four main categories; (iii) page 2 (clothing model) that represents the product code, composed of a letter concatenated to an integer number; (iv) location that describes the position of the product in the web page layout encoded for each part of the display (divided into six parts); and (v) order that reports the sequence of clicks during one session.
  4. The group of attributes related to the browsed product includes: (i) color, where colors of products are encoded as integers; (ii) price, expressed in dollars; (iii) the binary flag price 2 that defines whether the price of a specific product exceeds the average price for the entire product category; and (iv) model photography, describing whether the model is frontal or profile using a dicotomic flag.
- The second dataset (D2) used is called Clue-lds (Landauer et al., 2022a). This represents a collection of log data retrieved from interactions between users and a cloud storage solution over a 5-year period. Each collected event is characterized by a specific typology and the identifier of the user (with its role and network identifier) who performed it. The exact moment at which it is recorded and a series of user location information in the case of network identifier availability. Data are available in the study by Landauer et al. (2022b) in the form of a JSON object such that each first-level key represents a feature of the original data, which, in turn, could consist of a group of attributes. An example is reported in Listing 1.
  The meaning of first-level attributes can be described as follows: (i) params: this feature comprising a dictionary containing additional details about the event, which might encompass information (like the file access pathway); (ii) type: a categorical variable that describes the typology of the event, i.e., the action performed by the user; (iii) time: ISO-formatted timestamp of the event; (iv) id: a distinct integer identifier for the log sample; (v) uid: a string that could contain a user name or an IP address, depending on whether the event is related to a user or an entity. This variable is strictly related to the user identifier; (vi) role: string that defines the role of a user (e.g., management, sales, technical, etc.); (vii) isLocalIP: dicotomic flag, which expresses whether the user is identified as local (true) or external (false); (viii) location: a nested dictionary containing information on the geographical location of users, such as their address, city, and longitude.
- The third dataset (D3) consisted of synthetic samples and was released by the CERT Insider Threat Center (Glasser and Lindauer, 2013, 2016). Among the versions released, we considered r5.2 in our article. In this release, actions taken by an organization of 2,000 employees are simulated and recorded over a period of 18 months. The insider threat scenarios modeled are as follows: (i) data theft; (ii) intellectual property theft; and (iii) IT sabotage. This is achieved since the information provided characterizes the organizational structure and the generic user. Specifically, the information provided covers six distinct instances, i.e., (i) authentication-related events (logon/logoff); (ii) email transmissions; (iii) device interactions; (iv) file operations; (v) HTTP events; and (vi) psychometric score assigned to each employee. Each dataset file contains a set of specific variables for each category analyzed, as outlined in Table 2. For each instance, it is shown whether the feature is included (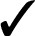) or not (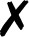). Note that in some cases, variables are shared between instances, but this does not imply sharing the interpretation of the evaluation of such a variable, because the semantic is instance-specific. For example, the activity of the logon (device) file can be logon/logoff(connect/disconnect).

Listing 1 **An example of sample in D2.**


 
       {‘‘params'': {‘‘path'': ‘‘/varied-tomato
                -muskox-engineer/profitable-copper-
                llama-classroomaide/appalling-purple
                -booby-qualitymanager''}, ‘‘type'':
                 ‘‘file_accessed'', ‘‘time'': ‘‘2022-
                04-12T08:26:56Z'', ‘‘uid'': ‘‘
                federal-jade-loon-handyman'', ‘‘id
                '': 49690432, ‘‘uidType'': ‘‘
                ipaddress'', ‘‘role'': null, ‘‘
                isLocalIP'': 0.0, ‘‘location'': {‘‘
                countryCode'': ‘‘IT'', ‘‘countryName
                '': ‘‘Italy'', ‘‘region'': ‘‘VE'',
                ‘‘city'': ‘‘San Don\u00e0 di Piave
                '', ‘‘latitude'': 45.6251, ‘‘
                longitude'': 12.5662, ‘‘timezone'':
                ‘‘Europe/Rome'', ‘‘postalCode'':
                ‘‘30027'', ‘‘metroCode'': null, ‘‘
                regionName'': ‘‘Venice'', ‘‘
                isInEuropeanUnion'': true, ‘‘
                continent'': ‘‘Europe'', ‘‘
                accuracyRadius'': 100}}
 

**Table 2:** D3 features per instance.

**Feature/file**	**Logon**	**Device**	**Email**	**http**	**File**	**Psychometric**
ID	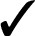	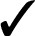	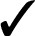	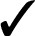	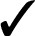	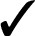
user	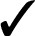	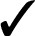	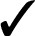	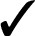	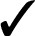	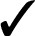
date	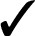	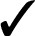	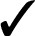	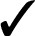	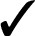	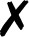
PC	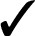	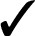	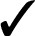	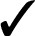	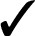	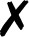
activity	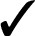	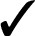	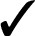	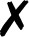	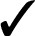	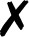
file_tree	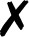	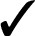	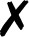	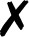	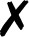	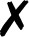
to	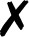	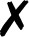	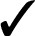	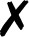	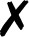	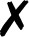
cc	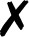	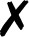	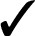	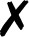	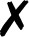	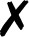
bcc	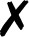	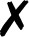	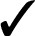	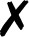	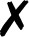	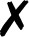
from	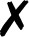	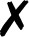	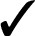	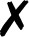	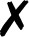	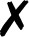
size	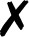	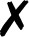	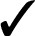	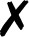	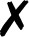	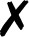
attachments	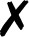	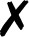	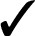	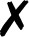	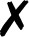	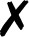
content	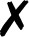	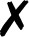	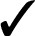	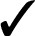	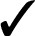	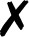
url	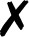	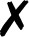	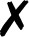	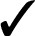	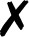	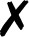
filename	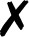	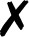	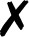	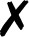	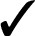	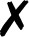
to_removable_media	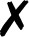	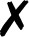	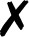	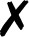	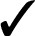	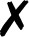
from_removable_media	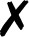	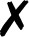	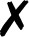	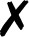	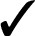	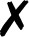
openness	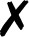	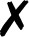	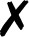	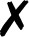	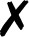	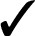
conscientiousness	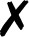	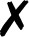	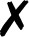	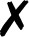	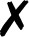	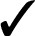
extraversion	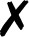	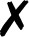	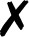	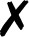	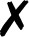	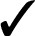
agreeableness	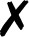	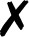	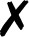	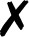	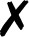	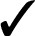
neuroticism	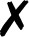	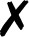	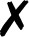	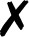	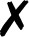	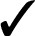

In Table 3, the main characteristics of the three datasets are listed. According to the problem at hand, there are both numeric and categorical data, i.e., our study considers mixed data. Note that although some of the selected clustering algorithms can work with mixed data (Ahmad and Khan, 2019), we will use appropriate strategies to transform categorical data into numerical data, as described below.

**Table 3:** Original data main characteristics.

**Dataset**	**No. samples**	**No. numeric (categorical) features**	**No. users**
D1	165,474	13 (1)	N/A^a^
D2	~5 × 10^7^	1 (8)	5389
D3	~8 × 10^7^	6 (27)	2 × 10^3^

^a^Not available.

#### 4.1.2 Pre-processing strategies

According to the information provided in Table 3, a different pre-processing strategy is required for each dataset. Specifically, each strategy adopted has the goal of accurately modeling real-life application contexts:

- D1 has a unique categorical column, that is, page 2 (clothing model). When analyzing such a column, it can be seen that it consists of a first letter followed by a number. Therefore, we divided this column into two new columns that are categorical and numerical, respectively. The generated categorical feature admits four possible values, i.e., A for 49,743 samples, P for 38,747 samples, C for 38,577 samples, and B for 38,407 samples. As a consequence, we applied a label encoder to map each letter to an integer number. Therefore, D1 is composed of 15 numerical features; thus, it is suitable for unsupervised learning methods, such as clustering algorithms. Furthermore, to reduce the impact due to the magnitude of some features, the *Z*-score strategy was used so that the data were normalized as $z=\frac{x-\mu }{\sigma }$, where μ and σ represent the mean and standard deviation of the original data, respectively. Therefore, unlike Datta et al. (2021), we did not apply One Hot Encoder (OHE) on page 2 (clothing model), avoiding explosion in the number of features. In fact, in the study by Datta et al. (2021), OHE resulted in ~70 features, making the need for principal component analysis (PCA) to reduce the number of columns to 15, obtaining an explained data variance equal to 85%. As a final point, we removed the column year because it assumes a unique value for all samples.
- To make the data suitable for the detection of misbehavior, we exploited the attack injection proposed by the authors of D2.^1^ It relies on the main idea of hijacking a user account by another one, i.e., a certain user's behavior patterns are changed to reflect someone else's from a given point in time. To implement such logic, two distinct users *u*_1_, *u*_2_ that interchange by means of the relative identifier are considered once a time, so that they meet the following properties: (i) both are operative until the instant *d*_*min*_ in which the switch occurs; (ii) after switching the identifiers, both users must be active for at least one day; (iii) only those who perform a minimum number of total events (*c*_*min*_) to which correspond a minimum number of unique event types (*a*_*min*_); and (iv) the considered patterns should not be too similar to be detected simply, but neither too different to make switching their identifiers irrelevant. All requirements are satisfied if the following set of equations holds (Landauer et al., 2022a):
(10) $$\{\begin{array}{l} |\{t_{i}\in d(u_{1(2)}):t_{i}<t_{s}\}|>d_{min}\\ c(u_{1(2)})>c_{min}\wedge |a(u_{1(2)})|>a_{min}\\ s_{min}<sim(u_{1},u_{2})=\omega_{1}\times \frac{\min(\frac{c(u_{1})}{\left|d(u_{1})\right|},\frac{c(u_{2})}{\left|d(u_{2})\right|})}{\max(\frac{c(u_{1})}{\left|d(u_{1})\right|},\frac{c(u_{2})}{\left|d(u_{2})\right|})}\\ +\omega_{2}\times \frac{\left|a(u_{1})\cap^{\text{​}}a(u_{2})\right|}{\left|a(u_{1})\cup^{\text{​}}a(u_{2})\right|}<s_{max} \end{array}$$
  where *d*(*u*_1(2)_) represents the number of days on which *u*_1_ and *u*_2_ generate an event, respectively, while *c*(*u*_1(2)_) and *a*(*u*_1(2)_) indicate the count, as well as all types of events generated by *u*_1_ and *u*_2_. Note that in Equation (10), *sim*(*u*_1_, *u*_2_) includes two weighting factors, i.e., ω_1_ and ω_2_ such that ω_1_+ω_2_ = 1. To implement attack hijacking, our setting follows the one proposed by the authors: *d*_*min*_ = 25 days; *c*_*min*_ = 100; *a*_*min*_ = 4; ω_1_ = 0.3; ω_2_ = 0.7; *s*_*min*_ = 0.1; *s*_*max*_ = 0.6. As a reasonable range from an application point of view, 6 months of random logs were considered in D2. Subsequently, the time column was divided into year, month, day, hours, minutes, and seconds. Analogously, the params column, which is an action-related dictionary, was split so that each key-value pair became a new feature of the dataset. The same logic was applied for location. Null values were padded with −1. Each categorical feature resulting from the splitting of params was transformed using a label encoder. As in D1, the entire dataset was normalized by applying a *Z*-score strategy.
- As a pre-processing strategy for D3, we used the one proposed in the study by Le et al. (2020).^2^ First, an employee context model is created consisting of information such as assigned assets, roles, work hours, authorizations, and relationships with colleagues. Given data in D3 and user contexts, the feature extraction phase starts aggregating data according to specific criteria, such as the observed period and the number of actions performed. Then, aggregated data are used to extract numerical vectors consisting of encoded categorical data and both frequency (number of resources access after a work hour, number of emails sent, etc.) and statistical (median, standard deviation of resource sizes, and the number of words in the websites accessed) features. Based on the aggregation condition, data are organized taking into account weeks, days, sessions, and subsessions. Specifically, in the last case, the user actions and duration of each session are indicated. As in the above pre-processing strategies, data are scaled through the *Z*-score.

The main characteristics of each pre-processed dataset are reported in Table 4. Furthermore, for each row, the Hopkins statistic is reported to quantify the cluster tendency of each dataset according to the following formula (Banerjee and Dave, 2004):


(11) $$\begin{array}{l} H=\frac{\overset{m_{b}}{\underset{l=1}{\sum }}r_{l}^{n}}{\overset{m_{b}}{\underset{l=1}{\sum }}r_{l}^{n}+\overset{m_{b}}{\underset{l=1}{\sum }}w_{l}^{n}} \end{array}$$

where in Equation (11): *m*_*b*_<*m*; *r*_*l*_ represents the minimum distance from a point sampled from a subspace of *X* to its closest pattern in X; *w*_*l*_ is the minimum distance from a randomly selected pattern in X to its nearest neighbor (*m*_*b*_ out of the available *m* patterns are marked at random for this purpose). Given such a construction, *H*∈[0, 1] and *H* → 0 indicates clustered data because *H* evaluates whether the data are generated by a *n*-variate uniform distribution on the hyper-rectangle with *n* sides of length equal to the *n* ranges of the original variables. From Table 4, all three datasets appear to be clusterable because the obtained *H* values are close enough to 0.

**Table 4:** Pre-processed data main characteristics and Hopkins statistic.

**Dataset**	**No. samples**	**No. numeric (categorical) features**	**No. users**	**Hopkins**
D1	165,474	14 (0)	N/A	4.59 × 10^−2^
D2	499,097	87 (0)	290	1.04 × 10^−4^
D3	132,124	1,096 (0)	2 × 10^3^	5.22 × 10^−5^

### 4.2 Metrics used

Since one of the contributions of this article is the refinement of the evaluation proposed in the study by Datta et al. (2021), we consider the same set of intrinsic measures, i.e., the Silhouette, Calinski-Harabasz (CH), and Davies Bouldin index (DBI) scores, to which we add the analysis of training time. The first three metrics belong to internal validation methods, i.e., their scores are computed without knowledge of the ground truth. The internal validation indices are mainly based on the concepts of cohesion and separation. The first is intended as a proximity measure of samples within a cluster. The second method provides a method for measuring the proximity between groups. Accordingly, the following equations hold (Palacio-Niño and Berzal, 2019):


(12) $$\begin{array}{l} Silhouette=\frac{1}{m}\overset{m}{\underset{i=1}{\sum }}\frac{b\left(i\right)-a\left(i\right)}{\max\left\{a\left(i\right),b\left(i\right)\right\}} \end{array}$$

(13) $$\begin{array}{l} CH=\frac{\overset{K}{\underset{k=1}{\sum }}m_{k}||c_{k}-c{||}^{2}}{\text{K}-1}\times \frac{m-K}{\overset{K}{\underset{k=1}{\sum }}\overset{m_{k}}{\underset{i=1}{\sum }}||x_{i}-c_{k}{||}^{2}} \end{array}$$

(14) $$\begin{array}{l} DBI=\frac{1}{K}\overset{K}{\underset{k,j=1}{\sum }}\underset{k\neq j}{\max}\left(\frac{\Delta X_{k}+\Delta X_{j}}{\delta \left(X_{k},X_{j}\right)}\right) \end{array}$$

where: (i) in Equation (12), $a\left(i\right)=\frac{1}{\left|C_{a}\right|}\underset{j\in C_{a},i\neq j}{\sum }d\left(i,j\right)$ is the average distance to all points in the same cluster, and $b\left(i\right)=\underset{C_{b}\neq C_{a}}{\min}\frac{1}{\left|C_{b}\right|}\underset{j\in C_{b},i\neq j}{\sum }d\left(i,j\right)$ represents the minimum average distance between the example and the examples contained in each group that does not contain the actual one; (ii) in Equation (13), *m*_*k*_ is the number of samples in the *k*-th cluster, and c is the global centroid; (iii) in Equation (14), Δ*X*_*k*_ is the intracluster distance within the cluster *k*, and δ(*X*_*k*_, *X*_*j*_) is the intercluster distance between the *k*−*th* and *j*−*th* clusters. Well-separated clusters are typically obtained for a Silhouette score [Equation (12) ∈(−1, 1)] close to 1 and for high (low) value of CH(DBI). Each metric is evaluated by performing an algorithm hyper-parameter tuning process. In particular, to the best of our knowledge, there are no other similar studies using D2 or D3. Moreover, when D1 is used, there is no evidence of a hyper-parameter configuration process. Our analysis was performed using the following reasonable ranges: (i) *K* = 2:1:20; (ii) ε = 0.2:0.2:3.8; (iii) *m*_*cluste*_*r*__*size*__ = 5:1:25; (iv) τ = 10:5:100; (v) *K*_*nn*_ = 5:5:100. Then, we considered the best configuration, which is achieved using the hyper-parameter simultaneously selected by the aforementioned methods (to which corresponds the best metric score the higher number of time). Given this setting, the number of samples within the predicted clusters, denoted with ρ, is used to perform a cluster density analysis. This can be exploited as in the study by Parwez et al. (2017) for anomaly detection; therefore, anomalies are considered clusters with fewer objects (if found).

### 4.3 Implementation details and hardware setting

According to the current development trend in the ML (Raschka et al., 2020) field, our comprehensive investigation was implemented in Python. Table 5 summarizes the implementation details of all algorithms compared, reporting the reference to the Python classical or custom package that provides the code. Moreover, the custom hyper-parameter setting is indicated for those that did not engage in the previously described tuning process. For example, we use the thumb rule used in the study by Datta et al. (2021) to set *m*_*pts*_ in DBSCAN (except for D2 and D3, where *m*_*pts*_ = 5), HDBSCAN, and OPTICS.

**Table 5:** Implementation details summary.

**Algorithm**	**Python package**	**Non-default hyper-parameters**
*K*-means		*K*
GMM		$K,reg_{\Sigma_{k}}=10^{-4}$ ^*a*^
DBSCAN	scikit-learn (Pedregosa et al., 2011)	ε, *m*_*pts*_ = 2 × *n*
OPTICS		*K* ^*b*^
Mini-Batch *K*-means		*K*
Fuzzy c-means	scikit-fda (Ramos-Carreño et al., 2023)	*K*
BIRCH	pyclustering (Novikov, 2019)	*K*
HDBSCAN	hdbscan (McInnes et al., 2017)	*m*_*cluste*_*r*__*size*__, *m*_*pts*_ = 2 × *n*
DenMune	pyMune (Abbas et al., 2023)	*K*_*nn*_
Scalable *K*-means++	Public repository^*c*^	*K*
Nested mini-batch *K*-means	Public repository^*d*^	*K*
SSC-OMP	Public repository^*e*^	*K*
EnSC		
		*K*, algorithm = spams^*f*^
FINCH	Public repository^*g*^	*K* ^*h*^
SCC	Public repository^*i*^	τ

^*a*^ Non-negative regularization factor ensuring Σ_*k*_ is positive for all *k*∈{1, ..., *K*}. ^*b*^ We tuned the number of clusters corresponding to the fuzzy c-means step. ^*c*^ https://github.com/SheliaXin/Scalable-K-means- (accessed October 10, 2023). ^*d*^ https://github.com/sharanry/Nested-K-Means (accessed October 10, 2023). ^*e*^ https://github.com/ChongYou/subspace-clustering (accessed October 10, 2023). ^*f*^ The sparse estimation is performed through the spams package: https://github.com/getspams/spams-python (accessed October 10, 2023). ^*g*^ https://github.com/ssarfraz/FINCH-Clustering (accessed October 10, 2023). ^*h*^ We also considered the case in which no number of clusters is required. ^*i*^ https://github.com/nmonath/scc/ (accessed October 10, 2023).

The visualization strategy of the results was inspired by the yellowbrick package (Bengfort and Bilbro, 2019). However, as shown in Table 5, not all algorithms were implemented using scikit-learn; therefore, we produced *ad hoc* Python visualization scripts that take advantage of matplotlib (Hunter, 2007) and seaborn (Waskom, 2021) graphical libraries. All experiments were performed on a workstation with an octa-core CPU and 64 GB RAM.

## 5 Results

### 5.1 Performance comparison

This section provides a performance comparison between the aforementioned metric trends with respect to the variation of the hyper-parameter setting for each algorithm. In particular, line plots were adopted for internal validation metrics, whereas training time was represented as a linear approximation of the actual trend, with a confidence interval of 95%. Note that we used a logarithmic scale for the CH measure because of its magnitude.

**Figure 1:**
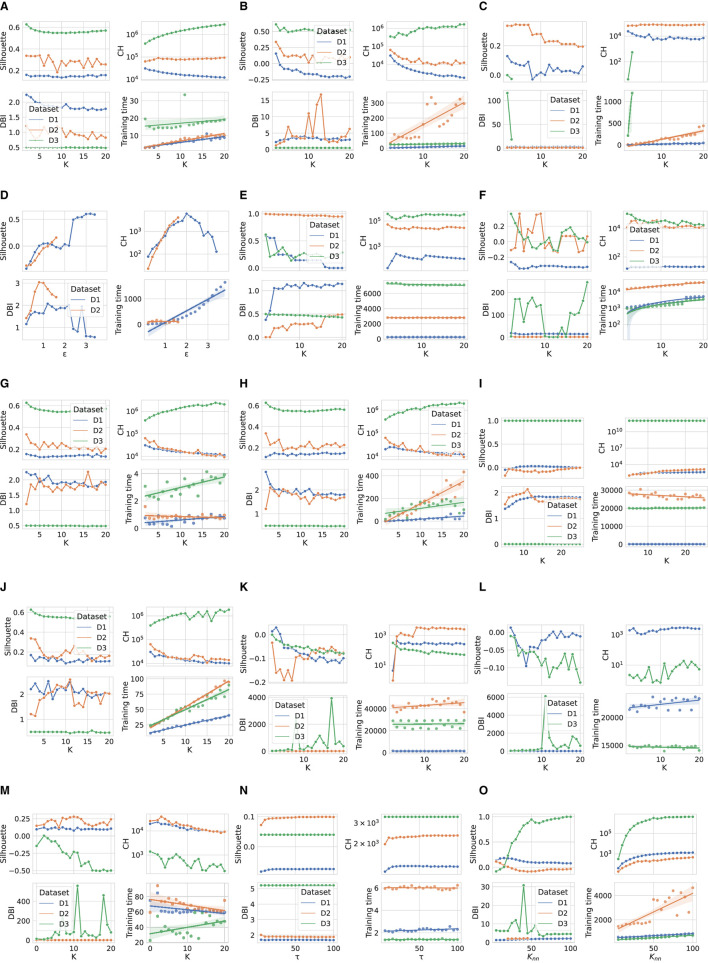
Performance comparison of evaluated clustering algorithms. **(A)** *K*-means. **(B)** Fuzzy c-means. **(C)** GMM. **(D)** DBSCAN. **(E)** BIRCH. **(F)** OPTICS. **(G)** Mini-Batch *K*-means. **(H)** Scalable *K*-means++. **(I)** HDBSCAN. **(J)** Nested mini-batch *K*-means. **(*K*)** SSC-OMP. **(L)** EnSC. **(M)** FINCH. **(N)** SCC. **(O)** DenMune.

Figure 1 shows the performance comparison analysis per algorithm. This can be described as follows:

- The results obtained for the *K*-means algorithm are grouped in Figure 1A. The Silhouette score reaches the maximum value for *K* = 2, for D1 and D3. As *K* increases, there is a degradation in the line generated by this metric for the third dataset, while a jagged curve is observed for D2 when *K*>5 (a spike is observed for *K* = 7). Taking into account D1, such a score tends to improve for *K* values close to 20. For this specific dataset, this result counters with the CH trend, which decreases as *K* increases. Meanwhile, CH achieves the maximum values for high *K* values when the datasets used are D2 and D3. The DBI is minimum for *K* = 4(16) in the case of D2(D1), while it follows an almost steady pattern for D3. Finally, the training time increases approximately linearly with *K* for all datasets.
- When examining the results achieved by the Fuzzy c-means algorithm (see Figure 1B), a performance decline is observed for all metric scores for D1 and D2 compared to the *K*-means experiment. Instead, the same results are obtained for the utmost case, which is again *K* = 2. When *K* increases, the DBI can reach very undesirable values. For example, in the case of D2, DBI>15 for *K* = 13. On the contrary, the results produced on the third dataset are almost exact replicas of those obtained using the *K*-means algorithm. Moreover, the Fuzzy c-means is more disadvantageous than *K*-means from the training time point of view, especially in the case of D2.
- Figure 1C displays the results obtained for the GMM algorithm. First, based on the configuration reported in Table 5, the algorithm was not run when a number of components greater than three was selected for D3, although we decreased *reg*_Σ_*k*__ with respect to its default setting. Furthermore, unlike the two algorithms above, promising CH and DBI trends are found for both D1 and D2, although *K* reaches higher values. With regard to Silhouette, it can be observed that the highest values are for *K*∈[2 − 7] for D1 and D2. Finally, taking into account the actual trend of the training time, one can infer that the GMM is influenced more by *n*, rather than *m*, when the algorithm searches for more components.
- Similar to GMM, the DBSCAN algorithm did not provide acceptable outcomes for D3, as for each value of ε tested, it assigned a cluster to each sample. For this reason, Figure 1D does not provide results for the third dataset. Furthermore, in the case of D2, the algorithm could not run for ε>1.6, with the step selected and the hardware setting used. However, close to the last valid experiments, both Silhouette and CH exhibit an increasing gradient, while DBI appears to decrease for ε>1. Nevertheless, in the case of D1, very relevant results are observed for both Silhouette and DBI with ε = 3.2, which, however, corresponds to a long training time. The latter increases almost linearly for ε>2.
- The BIRCH performance trends as a function of the number of clusters searched are presented in Figure 1E. First, training time appears to be unaffected by *K*. Note that the training time is the highest recorded so far for both D2 and D3, and in this case, the number of features is more likely to have a considerable impact on this overhead compared to the number of samples. Despite this effort, this algorithm appears to perform extremely promisingly, in fact: (i) for *K* = 2, very positive Silhouette scores are obtained for all datasets, and, especially, it is close to 1 for D2; (ii) for *K* = 2, the CH(DBI) assume (high) low values for D3(D1) and D2, while to *K* = 4(*K* = 20) corresponds the maximum (minimum) DBI for D1(D3).
- The OPTICS performance is represented in Figure 1F. For such an algorithm, *K* = 9 results in the highest Silhouette and DBI scores for the second dataset, respectively. The first-rate settings for D1 and D3 are given by *K* = 2 (maximum Silhouette and CH, and minimum training time). Regarding computational overhead, the overall training time is considerably long, especially when *m* is very high, as in the case of D2. As a final point, the training time grows nearly linearly with *K*.
- The performance of the Mini-batch *K*-means shown in Figure 1G is comparable to that obtained by the *K*-means algorithm in Figure 1A for low *K* values. In this case, *K* = 2 results in the highest Silhouette score. The main differences concern the CH and DBI curves, for which a performance drop is observed as the value of *K* increases. As expected, the main advantage with respect to the *K*-means is the shorter training time. Furthermore, another difference for this measure is given by the approximate linear trend in D2, which is descending for the mini-batch algorithm approach. This result is consistent with the working procedure of the algorithm, which reduces the overhead because of the mini-batch of samples used.
- Figure 1H shows the scores achieved by the metrics evaluated using the Scalable *K*-means++ algorithm. The observed trends are extremely similar to those of the *K*-means-based algorithms evaluated so far. However, a considerable difference is observed for the training time curves, which follows a linear increase with K and is longer than that of the *K*-means, especially when D2 is used.
- The HDBSCAN performance (Figure 1I) denotes a lack of efficiency of the algorithm on D1 and D2 datasets according to the metric scores achieved. On the other hand, the same set of graphs indicates that the algorithm exhibits very promising results when D3 is used, regardless of the value of *m*_*cluste*_*r*__*size*__. The training time trend of HDBSCAN highlights a significant overhead in the case of D2 and D3. However, it decreases with increasing *m*_*cluste*_*r*__*size*__ in experiments that involve the second dataset.
- The nested mini-batch *K*-means performs the same with respect to the *K*-means-based algorithms discussed above on the third dataset, as seen in Figure 1J. On the other hand, in the case of the D1 and D2 datasets, the values obtained from the DBI metric follow a more chaotic pattern as *K* increases. Similar to the trend observed for the previously mentioned algorithms, Silhouette and CH decreases with the increase of *K*. The training time grows linearly with such a hyper-parameter, with a more significant angular coefficient even in the case of the first dataset.
- According to the results provided in Figure 1K, the SSC-OMP algorithm yields in Silhouette values lower than zero for all evaluated datasets, expect for *K* = 2 and *K* = 3 when the first dataset is used. The CH increases (decreases) with *K* for D2(D1/D3). On the other hand, DBI assumes low values for both D1 and D2, regardless of *K*. On the contrary, it results in an unstable trend for D3, reaching very high values (e.g., ~4 × 10^3^ for *K* = 17). Finally, the computational overhead is significant, especially in the case of D2, where for each *K* value, a training time greater than 4 × 10^4^ s is observed.
- With regard to Figure 1L, the EnSC did not provide results on D2 using our data pre-processing strategy and hardware setup. Furthermore, the algorithm did not result in satisfactory performance. Specifically: (i) the Silhouette obtained is clipped in the range [−0.15, 0]; (ii) the CH follows a jagged trend that continuously oscillates between high and low values; and (iii) the DBI score is stable to low values for the first dataset and reaches an undesirable spike in the case of D3 for *K* = 11.
- The performance achieved by the FINCH algorithm is shown in Figure 1M. Note that *K* = 0 indicates that the algorithm was run without specifying the number of clusters to search for. The training time appears reasonable, even for large data (which appears to decrease linearly with increasing *K* when there are many samples). The CH decreases with the increase of *K*, regardless of the dataset considered. The DBI is almost stable for each *K*-value for the D1 and D2 datasets, while it assumes a skewed trend for D3. Regarding the Silhouette score, it is lower than zero (expect for *K* = 2) in the case of D3; on the contrary, it fluctuates around 0.1 and 0.2 for D1 and D2, respectively.
- The metric scores achieved by the SCC algorithm with respect to the variation of τ are displayed in Figure 1N. It shows a main finding, that is, the metrics are not affected by changes in τ, regardless of the dataset considered. In fact, except for the growth (descent) of Silhouette and Calinski (Davies) observed for τ < 15 in the case of D1 and D2, subsequent trends are almost stable. However, although SCC is advantageous from the point of view of training time, it appears to be ineffective from the rest of the scores.
- Figure 1O depicts the performance analysis for the DenMune algorithm. For D1 and D2, both Silhouette and CH scores are bounded in the ranges [0, 0.2] and [−0.35, 0], respectively; therefore, it results in unattractive performance for the first two datasets. Furthermore, in the D2 case, the DBI was logged merely for *K*_*nn*_∈[20, 45] without causing a memory segmentation error, because the calculation of this index is very expansive. Promising results are obtained for D3 when *K*_*nn*_>75. A key result is related to the trend in training time, which increases linearly with *K*_*nn*_ and appears lower for D3 than the other datasets.

**Figure 2:**
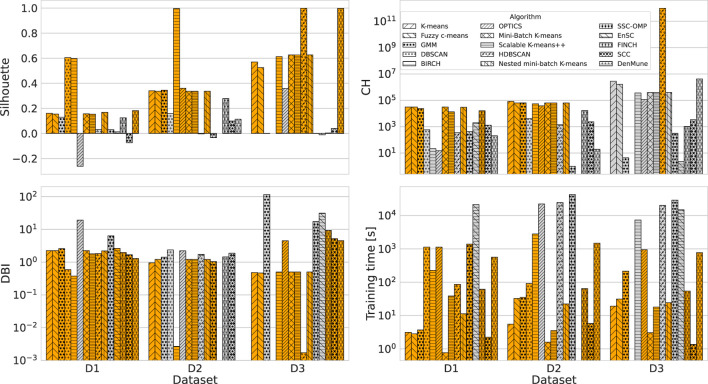
Top-performer algorithms achieving a score greater (in the cases of Silhouette and CH) or lower (in the cases of DBI and training time) than average.

Table 6 summarizes the configuration and metric score achieved by the top-performing algorithms. Recall that selection is done by considering the hyper-parameter that most frequently leads to the optimum value of the metrics evaluated. For example, the *K*-means algorithm for D3 reaches the maximum Silhouette for *K* = 2 and the minimum training time for *K* = 4. However, for *K* = 20, the maximum CH and the minimum DBI were found (see Figure 1A); therefore, the best configuration is *K* = 20. Note that in case there are several hyper-parameters with an equal selection frequency, the one that results in the highest value of Silhouette is chosen. We consider the latter metric to be the most relevant among the evaluated ones, as it incorporates both separation and cohesion to evaluate the performance of unsupervised learning algorithms (Palacio-Niño and Berzal, 2019). Furthermore, Figure 2 illustrates the performance of the clustering approaches per dataset using conditional coloring, so that the orange bars represent scores better than the average result achieved for the measure considered. It is possible to observe that in some cases, the frequency of algorithms showing better-than-average performance is influenced by edge cases (spikes or troughs). For example, in the case of D3, only one algorithm results in a CH value higher than the average, namely HDBSCAN. This denotes the performance dominance of this algorithm, a particular dataset (also orange bars for Silhouette and DBI), except for the training time. In addition, such a graphical representation is very useful for performing robustness analysis of the algorithms, i.e., to evaluate the capability of the algorithms to continue to produce good performance despite input data changes. In this regard, *K*-means (and derivatives) and fuzzy c-means are on average robust in about 92% of cases.

**Table 6:** Clustering metric scores achieved by optimally configured algorithms.

**Algorithm**	**Dataset**	**Hyper- parameter**	**Value**	**Silhouette**	**CH**	**DBI**	**Training time (s)**
*K*-means	D1	K	2	1.60 × 10^−1^	3.10 × 10^4^	2.24	3.14
	D2		7	3.40 × 10^−1^	8.36 × 10^4^	9.53 × 10^−1^	5.54
	D3		20	5.70 × 10^−1^	2.85 × 10^6^	4.80 × 10^−1^	19.11
Fuzzy c-means	D1	K	2	1.54 × 10^−1^	3.09 × 10^4^	2.25	2.84
	D2		2	3.36 × 10^−1^	6.36 × 10^4^	1.21	32.54
	D3		20	5.25 × 10^−1^	1.67 × 10^6^	4.66 × 10^−1^	31.17
GMM	D1	K	2	1.29 × 10^−1^	2.36 × 10^4^	2.58	3.72
	D2		4	3.45 × 10^−1^	6.45 × 10^4^	1.41	35.15
	D3		2	−5.76 × 10^−4^	4.39	1.15 × 10^2^	2.14 × 10^2^
DBSCAN	D1	ε	3.2	6.04 × 10^−1^	5.78 × 10^2^	5.93 × 10^−1^	1.12 × 10^3^
	D2		1.6	1.58 × 10^−1^	4.12 × 10^3^	2.37	91.67
	D3		None	None	None	None	None
BIRCH	D1	K	2	5.97 × 10^−1^	21.60	37.59	2.27 × 10^2^
	D2		2	9.96 × 10^−1^	5.38 × 10^4^	2.69 × 10^−3^	2.81 × 10^3^
	D3		2	6.13 × 10^−1^	3.61 × 10^5^	4.98 × 10^−1^	7.35 × 10^3^
OPTICS	D1	K	2	−2.62 × 10^−1^	15.02	19.14	1.12 × 10^3^
	D2		9	3.60 × 10^−1^	3.80 × 10^4^	2.21	2.22 × 10^4^
	D3		2	3.58 × 10^−1^	1.12 × 10^5^	4.50	9.44 × 10^2^
Mini-Batch *K*-means	D1	K	2	1.56 × 10^−1^	3.10 × 10^4^	2.25	7.70 × 10^−1^
	D2		2	3.36 × 10^−1^	6.36 × 10^4^	1.20	1.59
	D3		2	6.26 × 10^−1^	3.95 × 10^5^	5.00 × 10^−1^	3.06
Scalable *K*-means++	D1	*K*	16	1.53 × 10^−1^	1.34 × 10^4^	1.80	38.61
	D2	2	3.36 × 10^−1^	6.36 × 10^4^	1.20	3.55
	D3	2	6.26 × 10^−1^	3.95 × 10^5^	5.00 × 10^−1^	18.02
HDBSCAN	D1	*m*_*cluste*_*r*__*size*__	12	3.08 × 10^−2^	3.53 × 10^2^	1.82	86.43
	D2		25	−3.76 × 10^−3^	1.41 × 10^3^	1.73	2.45 × 10^4^
	D3		5	9.98 × 10^−1^	9.93 × 10^11^	1.71 × 10^−3^	2.00 × 10^4^
Nested	D1	K	2	1.68 × 10^−1^	3.03 × 10^4^	2.20	11.37
mini-batch	D2		2	3.36 × 10^−1^	6.36 × 10^4^	1.21	22.32
*K*-means	D3		2	6.26 × 10^−1^	3.95 × 10^5^	5.00 × 10^−1^	24.42
SSC-OMP	D1	K	3	2.96 × 10^−2^	4.20 × 10^2^	6.34	1.38 × 10^3^
	D2		2	−2.92 × 10^−2^	9.79 × 10^−1^	1.03	4.26 × 10^4^
	D3		2	−2.92 × 10^−4^	3.13 × 10^2^	17.39	2.89 × 10^4^
EnSC	D1	K	2	1.29 × 10^−2^	1.98 × 10^3^	2.63	2.14 × 10^4^
	D2		None	None	None	None	None
	D3		2	−1.07 × 10^−2^	2.19	31.26	1.49 × 10^4^
FINCH	D1	K	7	1.24 × 10^−1^	1.61 × 10^4^	1.44	61.36
	D2		10	2.78 × 10^−1^	1.68 × 10^4^	1.44	64.65
	D3		2	3.66 × 10^−3^	1.04 × 10^3^	9.28	54.37
SCC	D1	τ	70	−7.41 × 10^−2^	1.28 × 10^3^	1.69	2.23
	D2		80	9.94 × 10^−2^	2.34 × 10^3^	1.87	5.81
	D3		5	3.99 × 10^−2^	3.39 × 10^3^	5.21	1.36
DenMune	D1	*K*_*nn*_	15	1.82 × 10^−2^	2.03 × 10^2^	1.28	5.63 × 10^2^
	D2		5	1.13 × 10^−1^	19.09	None	1.45 × 10^3^
	D3		95	9.98 × 10^−1^	4.23 × 10^6^	4.53	7.72 × 10^2^

### 5.2 Cluster density analysis

**Figure 3:**
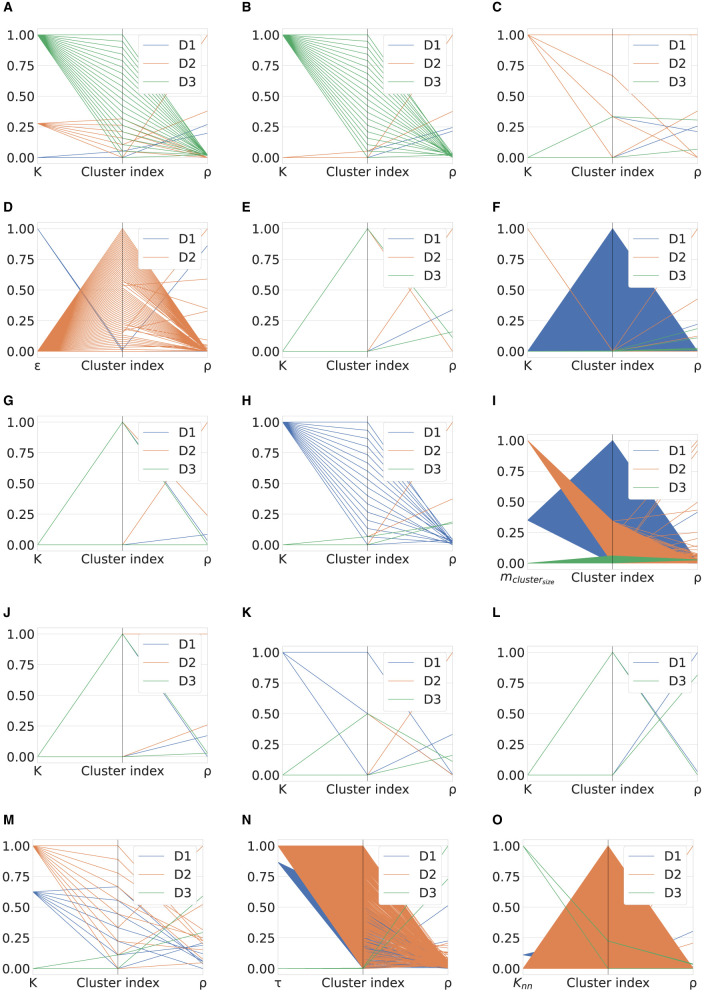
Cluster density analysis per algorithm. **(A)** *K*-means. **(B)** Fuzzy c-means. **(C)** GMM. **(D)** DBSCAN. **(E)** BIRCH. **(F)** OPTICS. **(G)** Mini-Batch *K*-means. **(H)** Scalable *K*-means++. **(I)** HDBSCAN. **(J)** Nested mini-batch *K*-means. **(*K*)** SSC-OMP. **(L)** EnSC. **(M)** FINCH. **(N)** SCC. **(O)** DenMune.

Cluster density analysis is performed on the algorithms listed in Table 6 using parallel coordinate plots. This type of graph comprises vertical lines. In our case, it is designed such that (i) the first line defines the top hyper-parameter setting; (ii) the second line includes the cluster indexes associated with the previous setting; and (iii) the third line shows the number of samples for each cluster index, i.e., the density ρ. This representation was selected for two main reasons: (i) each vertical line can represent a different physical measure; and (ii) parallel coordinate plots can effectively handle multidimensional measures. The complete analysis is shown in Figure 3 that includes 15 parallel coordinate plots (one for each algorithm). For visualization purposes to make the magnitudes of each measure comparable, we use a min-max normalization strategy so that each measure score is scaled in [0,1].

We use the notation ρ_*index*_ to indicate the number of samples corresponding to the *index*−*th* cluster, with *index*∈{0, *T*_*cf*_−1}, where *T*_*cf*_ represents the total cluster found (*T*_*cf*_ = *K*, when *K* is the hyper-parameter involved, except for OPTICS). The main insights of this analysis can be summarized as follows:

- Figure 3A illustrates the ρ distribution for the evaluated datasets according to the top configuration of the *K*-means algorithm. Specifically, in the case of D1, the algorithm generates two clusters with ρ_0_= 95,471 and ρ_1_= 70,003, respectively. On the other hand, for D2, two significant density peaks were found in the seven clusters found, that is, ρ_0_= 35,3146 and ρ_2_= 134,113, while all the remaining clusters contained fewer objects. Finally, the distribution of the samples for the 20 clusters generated in the case of the third dataset is balanced, in fact, ρ∈ [5,410, 7,780].
- The fuzzy c-means examination is presented in Figure 3B. Regarding D1, the results are very similar to those obtained for the *K*-means algorithm, in fact, ρ_0_= 77,544 and ρ_1_= 87,930. Similarly, in the case of D2, despite a lower *K*, the two inferred clusters have a distribution of samples very similar to the two notable among the seven found for *K*-means. Specifically, in this case, ρ_0_= 353,291 and ρ_1_= 134,155. Finally, despite the green patterns in Figures 3A, B appearing similar, in this case, the distribution range is more unbalanced than the previous, as ρ∈ [1,834, 12,560].
- As seen in Figure 3C, the GMM top configuration is achieved for two Gaussian components in the cases of D1 and D3. For the first dataset, the distribution of samples in the inferred clusters is similar to that obtained for the fuzzy c-means algorithm; in fact, ρ_0_= 90,934 and ρ_1_= 74,540. The two clusters created for D3 are unbalanced because one contains ~18% of the total number of samples. To be specific, ρ_0_= 24,088 and ρ_1_= 108,036. For the second dataset, optimal performance is achieved for *K* = 4, where ρ_0_= 134,154, ρ_1_ = 260, ρ_2_ = 1, and ρ_3_= 353,031.
- As reported in Figure 3D, the DBSCAN algorithm identifies two clusters for ε = 3.2 in the case of D1, with ρ_0_ = 55 and ρ_1_= 165,419, respectively. Meanwhile, *T*_*cf*_ = 61 for D2, of which four are characterized by a significant number of samples, i.e., ρ_9_= 191,835, ρ_32_= 113,033, ρ_34_= 66,640, and ρ_10_= 62,971.
- According to the results provided in Figure 3E, the inconsistent distribution of the samples can be observed for the two groups inferred for D1 and D2 by the BIRCH algorithm. In particular, two clusters consisting of only one sample and two samples were produced for these datasets. With regard to D3, the distribution of samples is slightly more unbalanced than that obtained using the algorithms that yield the two clusters examined so far, i.e., ρ_0_= 77,699 and ρ_1_= 54,425.
- The distribution of samples per cluster resulting from the application of the OPTICS algorithm is represented in Figure 3F. A complete triangular pattern was observed for D1 because 1923 clusters were generated. Among these, the first group includes 69512 samples, while the rest verifies ρ ≤ 145. On the other hand, five and eight clusters were obtained for D2 and D3, respectively. Once again, two main groups were found for the second (third) dataset with ρ_1_= 313,927 (ρ_4_= 58,175) and ρ_4_= 134,113 (ρ_0_= 34,597).
- The cluster density examination for Mini-Batch *K*-means is represented in Figure 3G. This confirms the results obtained for D1 using the *K*-means-based algorithms discussed above. In particular, ρ_0_ = 90, 096 and ρ_1_= 75,378 were found. For D2, the result obtained perfectly overlaps that obtained using fuzzy c-means. On the other hand, this algorithm configuration splits D3 into two well-balanced groups, i.e., ρ_0_= 65,717 and ρ_1_= 66,407.
- Figure 3H shows the evaluation of scalable *K*-means++. For the second dataset, this algorithm achieves the same result as the fuzzy c-means; therefore, ρ_0_= 353,291 and ρ_1_=134,155. Similarly, the same trend was observed for the third dataset. The main difference from the above-discussed *K*-means-based algorithms is the outcome obtained for D1. In this case, 16 groups were identified, so ρ_6_= 2,692 (ρ_7_= 19,313) is the smallest (highest) number of samples in a cluster.
- The cluster density analysis of the HDBSCAN algorithm reported in Figure 3I points out the following main findings per dataset: (i) *T*_*cf*_=1,164 clusters were identified for D1, with a dominant distribution for the first group, i.e., ρ_0_= 28,610, while ρ_*index*_ < 859 for *index* = 1, ..., *T*_*cf*_−1; (ii) in the case of the second dataset, *T*_*cf*_ = 400 and among these, the 1.75%(13.5%) groups contains more than 10^4^(10^3^) samples, i.e., most groups are composed of few samples; (iii) *T*_*cf*_ = 70 in correspondence of D3, with a well-balanced sample distribution for each cluster, as ρ∈ [1,746, 2,000].
- The nested mini-batch *K*-means cluster density analysis shown in Figure 3J provides the same results obtained for the mini-batch algorithm variant for the D2 and D3 datasets. In contrast, two clusters were generated from D1, with ρ_0_= 108,149 and ρ_1_= 57,325, respectively.
- Figure 3K depicts the SSC-OMP distribution of samples within the identified groups. The two clusters found for D2, consisting of ρ_0_= 487,443 and ρ_1_ = 3 samples. Instead, in the case of the third dataset, the distribution obtained is ρ_1_= 53,838 and ρ_0_= 78,286, which is slightly unbalanced compared with the others identified by the algorithms discussed above when searching for two clusters for the same dataset. As a final point, the first dataset is divided into three clusters, one of which is prominent with ρ_0_= 161,803.
- The results obtained for the EnSC algorithm (see Figure 3L) denote an unequal distribution of samples for both the D1 and D3 datasets in the two predicted clusters. In particular, ρ_0_= 160,629 (131,390) and ρ_1_= 4,845 (734) for D1 (D3).
- According to the results provided in Figure 3M, D1 samples are distributed among seven clusters with ρ∈ [11,926, 37,532]. The distribution of D3 samples is divided into two groups consisting of ρ_0_= 84,233 and ρ_1_ = 47, 891 samples. As a final consideration, the second dataset is divided into *T*_*cf*_ = 10 clusters, where the predominant one contains ρ_3_= 134,155 clusters.
- Figure 3N shows the distribution of samples within the groups inferred by the SCC algorithm. Consequently, the D1 dataset is divided into 138 groups, and these two groups are predominant, namely ρ_5_= 39,168 and ρ_20_= 17,192. The number of clusters produced doubled in the case of the second dataset. Specifically, 40 groups have more than 3 × 10^3^ samples and of these two are dominant, since they contain ρ_5_= 39,168 and 17,192 samples, respectively. Finally, in the case of D3, the samples are divided into two groups, comprising 57.8 and 42.2% of the total, separately.
- As seen in Figure 3O, the results obtained by the DenMune algorithm for such an analysis reveal that *T*_*cf*_= 5,103 (49,884) for D1 (D2). In both cases, the distribution of samples per cluster is unequal, since: (i) ρ_0_= 16,127 and ρ_*index*_ < 847 with 1 < *index*<*T*_*cf*_−1 for D1; and (ii) ρ_0_= 53,038, ρ_1_= 10,993 and ρ_*index*_ < 282 with 2 < *index*<*T*_*cf*_−1 for D2. For the third dataset, the cluster density examination is similar to that of HDBSCAN. In fact, *T*_*cf*_ = 71 with a well-balanced sample distribution for each group, as ρ∈ [1,759, 2,000], and only one cluster with 62 samples.

## 6 Discussion

**Figure 4:**
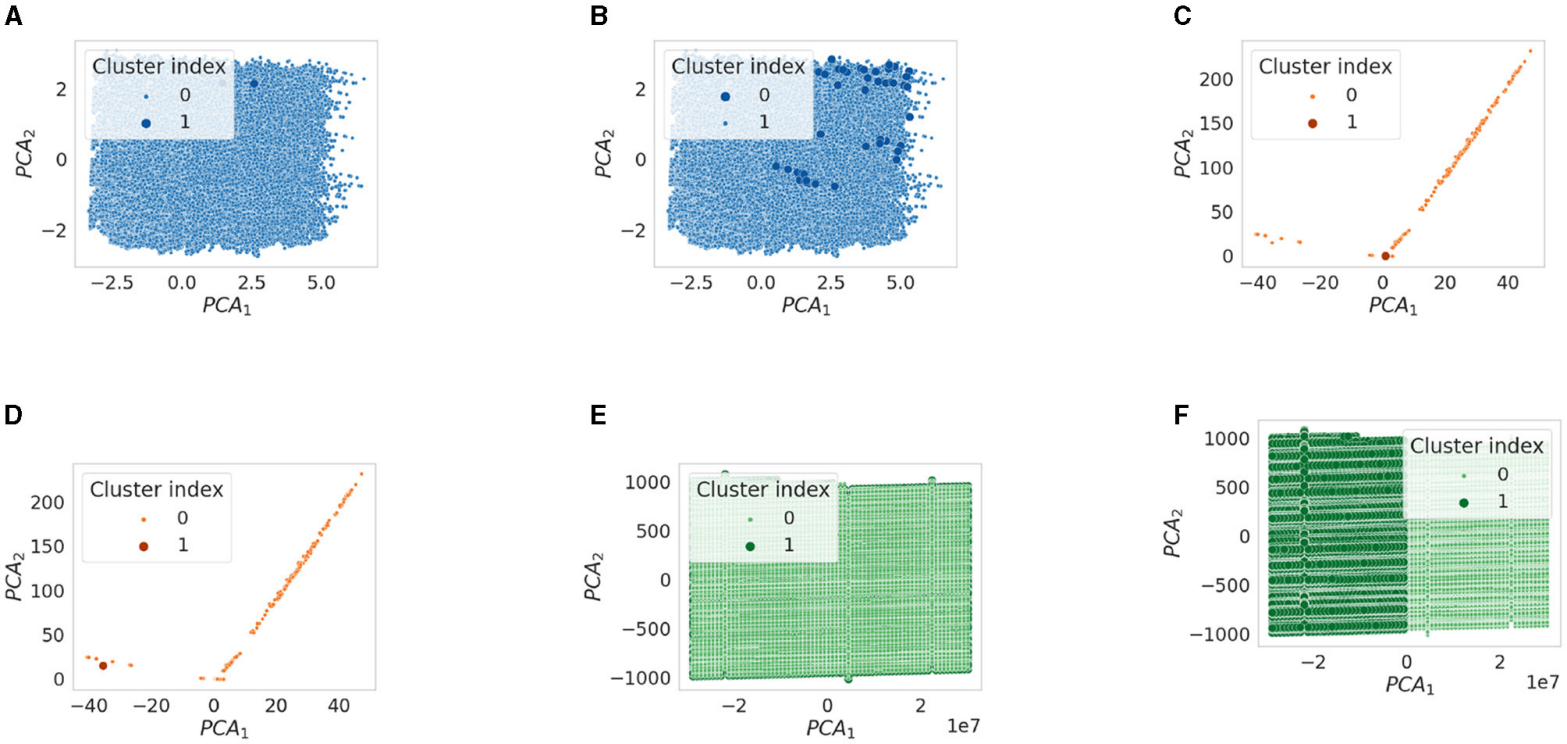
Analysis of points placement within the clusters inferred by some algorithms in a bi-dimensional space. **(A)** BIRCH (D1). **(B)** DBSCAN (D1). **(C)** SSC-OMP (D2). **(D)** BIRCH (D2). **(E)** GMM (D3). **(F)** Mini-Batch *K*-means (D3).

The main findings of our comprehensive study can be listed as follows:

- Two algorithms, namely BIRCH and DBSCAN, provided very promising results when using D1. In particular, the DBSCAN configuration we found in our work, combined with our pre-processing strategy, outperforms its counterpart in the study by Datta et al. (2021). The *K*-means-based algorithms, GMM, BIRCH, and FINCH, have shown promising performance on the D2 dataset. Given the volume of data, the clustering algorithms provided satisfactory outcomes for a set of real behaviors that were subsequently hijacked. As far as this dataset is concerned, the cluster density analysis showed in most cases the presence of two dominant clusters, even for a larger number of identified groups. The 50% of the algorithms evaluated yielded encouraging results for the third dataset. Among these, very positive results are obtained from HDBSCAN and DenMune, which divide the dataset into 70 groups, each of which contains a number of samples very close to the number of users in the dataset (see Table 4). As a consequence, combining the pre-processing strategy proposed in the study by Le et al. (2020) with clustering algorithms (e.g., one can set *K* = 70) can be a valuable approach to develop an effective UEBA engine using the CERT dataset, which, however, remains an artificial dataset.
- It was possible to realize that there is no direct relationship between the effectiveness highlighted by the metric scores and the number of samples per cluster inferred. We can assume that the metrics scores undergo considerable differences depending on which (not how many) samples are part of the identified groups. To support this thesis, we represented in a bidimensional space, obtained through the PCA, the distribution of samples in the clusters identified by some cases of interest. Specifically, we considered the two best-performing algorithms for D1, i.e., BIRCH and DBSCAN. Although their performance is comparable, the clusters differ in terms of the obtained densities and sample placement, as shown in Figures 4A, B. In addition, in the case of D2, we considered BIRCH (high-quality performance) and SSC-OMP (poor performance), both resulting in two groups, one of which is extremely dense and the other consists of a single sample. Figures 4C, D reveal that the different arrangement of a single sample can lead to a considerable change in the Silhouette score (see Table 6), which uses distances as the basis for scoring. As further evidence, in the case of the third dataset, a balanced distribution of samples within two inferred clusters leads to an overall benefit of metrics scores. This discovery is shown in Figures 4E, F, which compares the positioning of the samples in the two clusters found by the GMM (poor performance) and the Mini-batch *K*-means (positive performance achieved by each *K*-means-based algorithm with *K* = 2). In the absence of such a relationship, in some cases, such as BIRCH for D1 and D2, it would not be reliable to use clustering algorithms as anomaly detection systems based on cluster density analysis, as in the study by Parwez et al. (2017). For such purposes, an external evaluation methodology would be more appropriate, net of ground-truth knowledge (Palacio-Niño and Berzal, 2019).

## 7 Clustering algorithms feasibility for User and Entity Behavior Analytics

Clustering algorithms can be applied within UEBA systems to group similar behaviors or entities together based on various features or attributes. However, our investigation shows that the reliability of clustering algorithms in UEBA depends on several factors:

- Data volume: UEBA systems deal with large amounts of data from various sources, such as network logs, user activity, and application logs. The challenge lies in effectively processing and analyzing these data in real-time. Clustering algorithms must be scalable and efficient to handle the large volume and complexity of data generated by modern IT systems. To the best of our knowledge, our study is the first to consider this factor in such an investigation. Among the scalable algorithms evaluated, the variants of the *K*-means algorithm and BIRCH result in an acceptable trade-off between performance and capability in handling large data with a low execution time. In contrast, there is no evidence of satisfactory performance from emerging scalable subspace-type algorithms. Furthermore, according to the hardware architecture and software configurations used, we observed that not all clustering approaches could handle large amounts of data, and this was verified for both popular (DBSCAN on D3) and emerging (EnSC on D2) algorithms.
- Feature engineering and selection: The choice of data pre-processing strategy used to represent user or entity behavior is crucial. Identifying relevant features that characterize normal and abnormal behavior can be challenging, especially in dynamic environments where behaviors evolve over time, amplifying the concept drift effect of the data. Effective feature selection can improve the performance of some clustering algorithms by capturing relevant patterns in the data. In this regard, a slight difference in the pre-processing strategy adopted by us and Datta et al. (2021) in D1 results in an evident deviation of the algorithm performance. Although our approach left the total variance of the data unchanged, the most classical clustering algorithm, namely *K*-means, benefits more from the pre-processing proposed by the study by Datta et al. (2021). In this case, it might be preferable to use dummy variables to encode categorical variables. On the contrary, DBSCAN gains from our data elaboration method. Meanwhile, the feature engineering method proposed by Le et al. (2020) for D3 makes the data very suitable for the HDBSCAN and DenMune algorithms, so they can achieve very promising performance, although this benefit is not observed for all approaches. As a final point of discussion, in the case of the second dataset, some relevant variables can most likely be identified by experts in the cloud communications domain.
- Domain-specific considerations: The effectiveness of clustering algorithms in UEBA also depends on the specific domain and context in which they are applied. Different use cases, emulated in this study using three different datasets, require tailored approaches to user and entity behavior analysis. For example, the effectiveness of the HDBSCAN and DenMune is valid only for the domain modeled by the D3 datasets, whereas they do not seem suitable for operation in real use cases, such as D2.
- Explainability: From a cybersecurity perspective, it is essential to interpret and explain the reasoning behind the results of ML algorithms to facilitate effective detection and response to threats. The user abnormal behavior detection strategy used in the study by Parwez et al. (2017) appears to be not suitable for the UEBA use cases considered in our investigation, as shown by cluster density analysis, because each algorithm produces different results. Interpreting a sparse cluster as anomalous cannot be considered true as a general rule. In addition, as shown in our study, the variation in performance is sensitive to the adjustments in the hyper-parameters of the algorithm. Furthermore, by varying the algorithmic setting, the implemented anomaly detection strategy also changes; therefore, it is not straightforward to infer that clustering algorithms can actually operate as real-time detection tools, as stated in the study by Datta et al. (2021). The explainability can benefit from combining several analyses, as was done in our article that showed how the HDBSCAN and DenMune algorithms were able to segment the dataset into user behaviors so that each group consists of statistical units close to the number of users in the dataset.

The comprehensive analysis proposed in our study highlighted the existence of a gap between the validation of clustering algorithms in proof-of-concept experiments and their deployment as UEBA engines. The critical outcomes provided by our investigation indicate that to allow clustering algorithms to actually be employed in real use cases, further investigation is required to highlight the strengths and critical aspects of these methodologies.

## 8 Conclusion and future work

The challenge of UEBA is gaining attention in the cybersecurity scientific community, as cyber threats often stem from human actions. With the rapid advancement of ML, systems capable of detecting insider threats from user behavior datasets have emerged. However, it is crucial to thoroughly examine the reliability of these techniques before applying them in the cybersecurity field. This study extensively evaluated the performance of a specific type of approach within the unsupervised learning paradigm, i.e., clustering algorithms. For this purpose, we used three datasets from the existing literature. Our study encompassed 15 clustering algorithms, ranging from the most traditional to the most recent. The experimental phase involved analyzing the trends of tailored metrics as the value of a characteristic hyper-parameter of the evaluated algorithm varied. In addition, we examined the density of the samples within the inferred clusters. A key finding was the partitioning of the CERT dataset achieved by HBDSCAN and DenMune. The resulting groups had a density very similar to the number of users in the data. While clustering algorithms provide the great advantage of grouping unlabeled data, making them suitable for discovering anomalous not-known-in-advance user and entity behaviors, validating these methodologies in proof-of-concept experiments can lead to approaches that hardly generalize to complex scenarios without rigorously considering factors such as feature engineering, domain constraints and specifications, the size of data involved, and the interpretability of the results obtained. In the context of future research endeavors, a significant avenue to explore involves conducting an ablation study on top-performing models, with a particular emphasis on the second dataset. In particular, its composition includes real-world user behavior collected from various anonymized users of a cloud sharing platform, which can be seamlessly blended to replicate scenarios of account hijacking, presenting a novel opportunity to tailor clustering algorithms specifically for anomaly detection applications. Furthermore, online clustering algorithms, which can update clusters in real-time or periodically re-cluster the data, will be valued for UEBA applications, because it is essential to satisfy the requirement of adaptability to dynamic environments.

## Data availability statement

Publicly available datasets were analyzed in this study. This data can be found here: D1-link: https://doi.org/10.24432/C5QK7X, name: clickstream data for online shopping, an (DOI): 10.24432/C5QK7X; D2-link: https://zenodo.org/records/7119953, name: Cloud-based User Entity Behavior Analytics Log Data Set, an (DOI): 10.5281/zenodo.7119953; D3-link: https://insights.sei.cmu.edu/library/insider-threat-test-dataset/, name: Insider Threat Test Dataset, an (DOI): 10.1184/R1/12841247.v1.

## Author contributions

PA: Conceptualization, Data curation, Resources, Software, Supervision, Validation, Visualization, Writing—review & editing. AMac: Conceptualization, Formal analysis, Investigation, Methodology, Validation, Visualization, Writing—original draft, Writing—review & editing. AMag: Data curation, Methodology, Software, Visualization, Writing—review & editing.

## Footnotes

^1^https://github.com/ait-aecid/clue-lds (accessed October 16, 2023).

^2^https://github.com/lcd-dal/feature-extraction-for-CERT-insider-threat-test-datasets (accessed October 3, 2023).
